# Microtubule forces drive nuclear damage in LMNA cardiomyopathy

**DOI:** 10.1101/2024.02.10.579774

**Published:** 2024-06-21

**Authors:** Daria Amiad Pavlov, Carmen Suay Corredera, Mohammad Dehghany, Julie Heffler, Kaitlyn M. Shen, Noam Zuela-Sopilniak, Rani Randell, Keita Uchida, Rajan Jain, Vivek Shenoy, Jan Lammerding, Benjamin Prosser

**Affiliations:** 1 Department of Physiology, Pennsylvania Muscle Institute, Perelman School of Medicine, University of Pennsylvania; 2 Weill Institute for Cell & Molecular Biology, Cornell University; 3 Department of Materials Science and Engineering, Center for Engineering Mechanobiology, University of Pennsylvania; 4 Departments of Medicine and Cell and Developmental Biology, Penn Cardiovascular Institute, Penn Epigenetics Institute, Perelman School of Medicine, University of Pennsylvania

## Abstract

Nuclear homeostasis requires a balance of forces between the cytoskeleton and nucleus. Variants in *LMNA* disrupt this balance by weakening the nuclear lamina, resulting in nuclear damage in contractile tissues and ultimately muscle disease. Intriguingly, disrupting the LINC complex that connects the cytoskeleton to the nucleus has emerged as a promising strategy to ameliorate *LMNA* cardiomyopathy. Yet how LINC disruption protects the cardiomyocyte nucleus remains unclear. To address this, we developed an assay to quantify the coupling of cardiomyocyte contraction to nuclear deformation and interrogated its dependence on the lamina and LINC complex. We found that the LINC complex was surprisingly dispensable for transferring the majority of contractile strain into the nucleus, and that increased nuclear strain in *Lmna-*deficient myocytes was not rescued by LINC disruption. However, LINC disruption eliminated the cage of microtubules encircling the nucleus, and disrupting microtubules was sufficient to prevent nuclear damage induced by *LMNA* deficiency. Through computational modeling we simulated the mechanical stress fields surrounding cardiomyocyte nuclei and show how microtubule compression exploits local vulnerabilities to damage *LMNA*-deficient nuclei. Our work pinpoints localized, microtubule-dependent force transmission through the LINC complex as a pathological driver and therapeutic target for *LMNA* cardiomyopathy.

## Graphical abstract

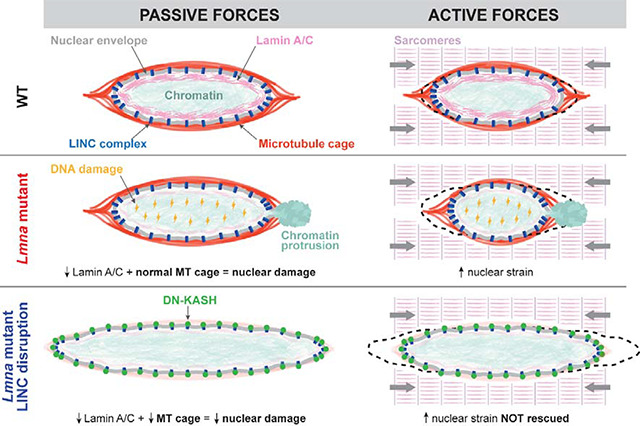

## Introduction

Mature cardiac muscle experiences an extreme mechanical environment as it continuously undergoes dynamic contraction and relaxation cycles. Accordingly, the cytoskeletal and nucleoskeletal architecture uniquely adapts to withstand and sense the mechanical load. In the adult cardiomyocyte, the actin-myosin, microtubule (MT) and desmin intermediate filament networks are physically coupled to the nucleus via the Linker of Nucleoskeleton and Cytoskeleton (LINC) complex that spans the nuclear envelope (NE). The LINC complex in turn interacts directly with the nuclear lamina, a meshwork of A and B-type lamin filaments that provides nuclear structural support and contributes to spatial chromatin organization. The LINC complex consists of various Nuclear Envelope Spectrin Repeat (nesprin) protein isoforms with conserved Klarsicht/ANC-1/Syne Homology (KASH) domains targeted to the outer nuclear membrane. In the intermembrane space the KASH domain interacts with Sad1p/UNC-84 domain-containing proteins SUN1 or SUN2, which in turn span the inner nuclear membrane to interact with the nuclear lamina and chromatin ^1,2^. Thus, nuclear homeostasis and integrity are dependent on the balance of cytoskeletal forces, nuclear resistance, and their proper coupling ^3^.

The importance of nuclear mechanical balance in the heart is demonstrated by human mutations in genes encoding lamins or other NE components that disproportionally affect highly contractile muscle tissues ^4,5^. Specifically, mutations in the *LMNA* gene that encodes A-type nuclear lamins cause a heterogenous group of diseases (“laminopathies”) with a prevalence of cardiomyopathies ^6^. The *LMNA* N195K missense mutation is associated with dilated cardiomyopathy (DCM) in patients, and the *Lmna*^N195K/N195K^ mouse model leads to heart failure and death within 12 weeks of age ^7^. Specific and effective treatments are not available for *LMNA* associated DCM, and the molecular mechanism of disease pathogenesis is poorly understood. A prevailing hypothesis points to mechanically induced structural damage to the NE when the lamina is compromised. This is based on evidence from lamin deficient and mutated models that show altered nuclear deformation, nuclear rupture, and impaired nuclear mechano-transduction ^8–13^.

Importantly, decoupling the nucleus from the cytoskeleton by disrupting the LINC complex restores cardiac function and extends the lifespan of *Lmna* associated DCM mice ^14,15^. While both actomyosin contractility ^12^ and MT mediated forces ^8,15^ have been suggested to drive nuclear damage in *Lmna* mutant models, the specific cytoskeletal forces responsible for nuclear damage and the mechanism of protection by LINC complex disruption remain unclear. This is in part due to the lack of direct tools to measure how cytoskeletal forces are transferred to the nucleus in the relevant physiological and mechanical environment ^4,5,12^. Adult cardiomyocytes are often binucleated with nuclei equally spaced in the center of the cardiomyocyte, surrounded by a dense sarcomeric network and perinuclear cage of MTs. This central location prevents direct physical access to probe nuclear stiffness, as often reported in non-mature cell lines or skeletal muscle fibers with peripheral nuclei ^16^.

Here we introduce a new assay to quantify sarcomere-nuclear strain coupling in contracting, adult cardiomyocytes. We use this assay to decipher the role of the LINC complex and the cytoskeleton in driving nuclear strain during active contraction. We further investigate mechanisms of nuclear damage in *Lmna* induced DCM, and protection from nuclear damage by cardiac specific LINC disruption ^17^. Surprisingly, we find that sarcomere-nuclear strain coupling is largely preserved upon disruption of the LINC complex. Further, LINC disruption does not reduce active nuclear strain in *Lmna*-deficient cardiomyocytes, suggesting that reduced nuclear strain during contraction is insufficient to explain protection from damage. Instead, we observe that LINC complex disruption eliminates the dense cage of MTs typically formed around the nuclei of cardiomyocytes, which reduces MT compressive forces at the nuclear tips and confers protection from nuclear rupture at these sites. We validate the role of MT compressive forces in *LMNA* nuclear damage by experimental disruption of the MT network in lamin A/C-deficient cardiomyocytes of mouse and human origin, and via a computational model that simulates the distribution of forces and sites of nuclear vulnerability in the cardiomyocyte.

## Results

### Active sarcomere-nuclear strain coupling in adult beating cardiomyocytes.

**Fig. 1:**
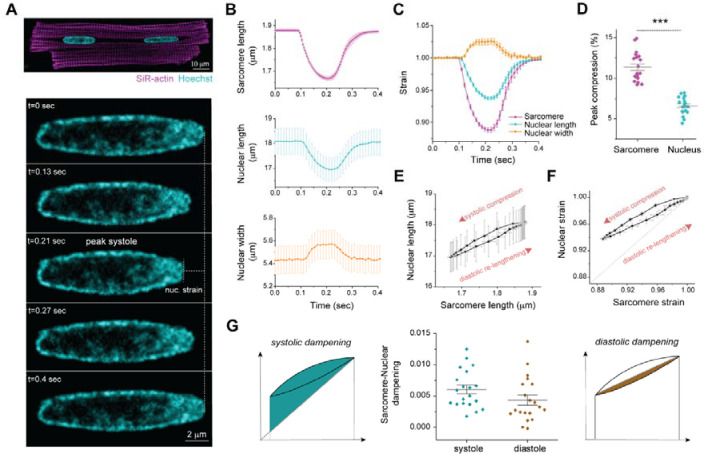
Active sarcomere-nuclear strain coupling in beating cardiomyocytes. (A) Live isolated adult rat cardiomyocyte stained with SiR-actin and Hoechst to visualize sarcomeres and DNA, respectively (top). Cardiomyocytes were stimulated at 1 Hz, and the nucleus imaged at 90 fps to follow nuclear deformation during the contraction cycle (bottom – representative time lapse images, see Supplementary movie S1). (B) Sarcomere length, nuclear length, and nuclear width recordings over time for a single contraction cycle. (C) Sarcomere strain, nuclear length strain and nuclear width strain over time. (D) Quantification of peak sarcomere and nuclear compression. (E) Sarcomere-nuclear coupling represented with a plot of nuclear length versus sarcomere length and (F) respective nucleus strain versus sarcomere strain. The latter, dimensionless strain coupling map depicts the dampened strain on the nucleus during systolic compression and diastolic re-lengthening, as marked by the deviation from a linear correlation (dotted line). (G) Sarcomere-nuclear strain dampening during systole quantified from area above linear correlation (left schematic). Diastolic dampening is quantified from area under end systolic linear correlation (right schematic). Data presented as mean ± standard error (SE) for 20 cells from a single representative adult rat heart. Statistical significance determined by two-tailed t-test (***, *p* < 0.001).

Measuring active mechanical signal transfer into the nucleus of mature cardiomyocytes is a missing component of interrogations into cardiac mechanobiology and *LMNA* driven cardiomyopathies, as the rapid contraction and central location of the nucleus pose technical challenges. Thus, we developed a method to quantify active sarcomere-nuclear strain coupling during electrically stimulated contractions in adult cardiomyocytes (Supplementary movie 1). We combined real-time measurements of sarcomere length change proximal to the nucleus with high spatiotemporal resolution (90 fps) Airyscan 2D imaging of the nucleus during steady-state contraction (Fig. 1A). Fig. 1B demonstrates that during the contraction cycle, sarcomere shortening and relaxation (magenta) are tightly coupled to the shortening and relaxation of nuclear length (cyan), with an opposing increase in nuclear width (orange). Notably, the relative change in sarcomere length, or strain, during the contraction cycle is not fully transferred into the nucleus (Fig. 1C). At peak systole, for 11.4 ± 0.4 % sarcomere compression, nuclear length compresses only 6.6 ± 0.3 % (Fig. 1D). We generated sarcomere-nuclear strain coupling maps by plotting the absolute sarcomere length versus the absolute nuclear length during systolic compression and diastolic re-lengthening (Fig. 1E), or the respective sarcomere length strain versus nuclear length strain (Fig. 1F). From here on ‘nuclear strain’ will be used to describe the nuclear strain along the contractile axis. This dimensionless representation provides improved visual comparison of the nuclear compression and re-lengthening in response to sarcomere shortening and relaxation. The dampening of the active strain on the nucleus is represented here by the upward deviation of the curve from the linear correlation (dotted line) as the sarcomere shortens and re-lengthens. We can further separate and quantify sarcomere-nuclear strain dampening during the systolic and diastolic phases of the contractile cycle (Fig. 1G). We quantify systolic dampening by integrating the area under the curve during contraction with respect to the linear relationship between sarcomere and nuclear strain (Fig. 1G, left). We isolate diastolic dampening by integrating the area under the curve during re-lengthening with respect to a linear relationship with intercept fixed at the end-systolic strain (Fig. 1G, right). With this approach we can dissect how specific perturbations may compromise coupling between the sarcomere and nucleus during each phase of the contractile cycle.

**Fig. 2:**
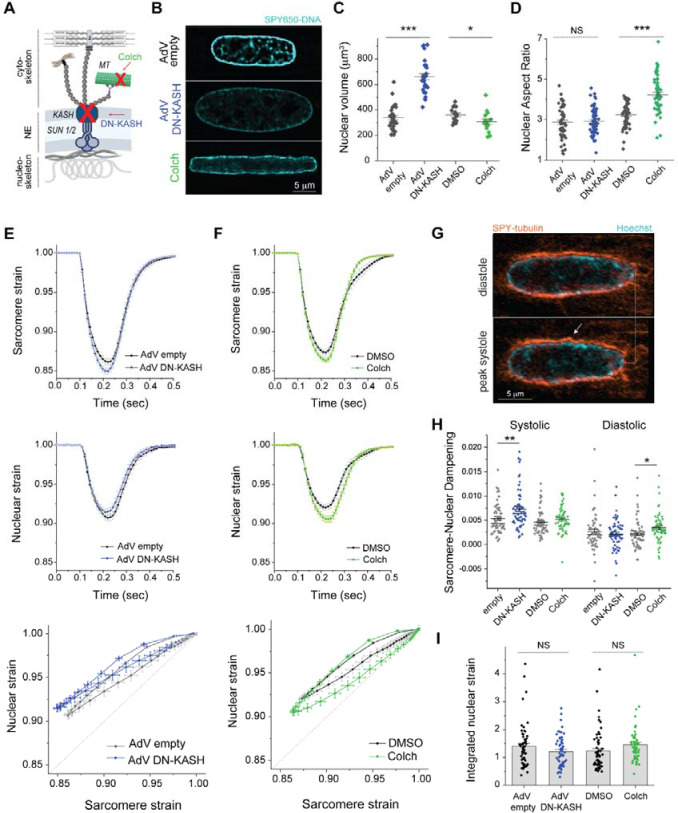
Distinct effects of LINC complex disruption and MT depolymerization on nuclear morphology and active sarcomere-nuclear strain coupling. (A) Schematic of cytoskeletal to nucleoskeletal connections and the experimental perturbations. (B) Live 3D super-resolution imaging of isolated adult rat cardiomyocyte nuclei (mid-nuclear planes are displayed). Nuclear volume (C) and aspect ratio (D) measurements following AdV DN-KASH or colchicine treatment. For panel (C): AdV empty and AdV DN-KASH (48 h): *N* = 3, *n* = 30, DMSO and colch (24 h): *N* = 2, *n* = 16. For panel (D): AdV empty and AdV DN-KASH (48 h): *N* = 3, *n* = 51, DMSO and colch (24h): *N* = 3, *n* = 51. Sarcomere strain (top) and nuclear strain (middle) over time, and sarcomere-nuclear stain coupling (bottom) for (E) AdV DN-KASH and (F) colchicine compared to their respective controls. (G) Snapshots of live, WT cardiomyocyte labeled with SPY-555 tubulin and Hoechst during diastole and peak systole demonstrating MT cage buckling (arrow) during contraction. (H) Quantification of sarcomere-nuclear strain dampening during the systolic and diastolic phases, for the indicated perturbations (see schematics in Fig. 1G). (I) Integrated nuclear strain over time during the contractile cycle for the indicated perturbations. AdV empty and AdV DN-KASH (48 h): *N* = 3, *n* = 51, DMSO and colch (24 h): *N* = 3, *n* = 51. Data presented as mean ± SE. Statistical significance determined by two-tailed t-test (*, *p* < 0.05; **, *p* < 0.01; ***, *p* < 0.001).

We next interrogated how cytoskeletal connections to the nucleus regulate sarcomere-nuclear strain coupling during active contraction. To disrupt all cytoskeletal interactions with the LINC complex, we utilized a previously validated adenovirus over-expressing a dominant-negative KASH peptide (AdV DN-KASH) which prevents interactions between endogenous nesprin and SUN proteins ^1,3^. In addition, we used colchicine (colch, 1 μM, overnight) to specifically eliminate the compressive forces exerted on the nucleus by the perinuclear MT cage (Fig. 2A). After 48 h adenoviral transduction of isolated rat cardiomyocytes, we confirmed that AdV DN-KASH disrupted the LINC complex through loss of perinuclear nesprin 1 (Supplementary Fig. S1A), and that 24 h colchicine resulted in a loss of MTs (Supplementary Fig. S1B). Neither acute LINC complex disruption nor MT depolymerization overtly altered sarcomere organization around the nucleus (Supplementary Fig. S1A–B) or the resting sarcomere length (Supplementary Fig. S1C).

Live, 3D super-resolution confocal imaging (Airyscan jDCV) of quiescent cardiomyocytes demonstrated distinct changes in resting nuclear morphology in response to both perturbations (Fig. 2B). Adult rat cardiomyocytes transduced with AdV DN-KASH resulted in increased nuclear size in 3 dimensions, with no change in nuclear aspect ratio but a substantial increase in nuclear volume (Fig. 2C). Conversely, colchicine treated nuclei displayed elongated morphology with increased nuclear aspect ratio and a subtle decrease in nuclear volume (Fig. 2C–D), in agreement with our earlier report ^3^.

Upon electrical excitation, peak sarcomere contractility increased subtly with AdV DN-KASH, yet peak nuclear compression slightly decreased (Fig. 2E, top and middle, Supplementary Fig. S1C, left). This indicates a modest yet significant decrease in sarcomere-nuclear strain coupling during myocyte contraction, as evident from the upward shift of the AdV DN-KASH curve in the strain coupling map (Fig. 2E bottom), and the significantly increased sarcomere-nuclear dampening during systole (Fig. 2H).

Colchicine treatment moderately increased sarcomere contractility and accelerated relaxation (Fig. 2F, top), consistent with microtubules providing a viscoelastic resistance to myocyte contraction ^18^. Yet, in contrast to AdV DN-KASH, nuclear strain was also increased, and nuclear relaxation was prolonged (Fig. 2F, top and middle, Supplementary Fig. 1C, right). These changes did not result in an overall upward or downward shift in the strain coupling curve, but rather increased area within the diastolic curve, or hysteresis (Fig. 2F, bottom). The prolonged nuclear relaxation upon MT depolymerization suggests that buckling of the MT cage during systole (Fig. 2G, Supplementary movie 2) might provide restoring force to facilitate nuclear relaxation during diastole ^19^. This is supported by increased sarcomere-nuclear dampening specifically during diastole in colchicine treated cardiomyocytes (Fig. 2H). To examine the cumulative strain on the nucleus during the full contractile cycle, we integrated the nuclear strain curve over time, and found no statistically significant differences between control and LINC complex or MT perturbed cells (Fig. 2I). Together, these results demonstrate that acute in vitro LINC complex disruption subtly reduces sarcomere-nuclear strain coupling, while nuclear re-lengthening is prolonged in the absence of MTs. They also highlight that most of the nuclear strain during active contraction is driven by the shortening and re-lengthening of nearby sarcomeres independent of connectivity via the LINC complex.

### Cardiac specific in-vivo disruption of the LINC complex protects from nuclear damage in lamin-deficient cardiomyocytes.

**Fig. 3:**
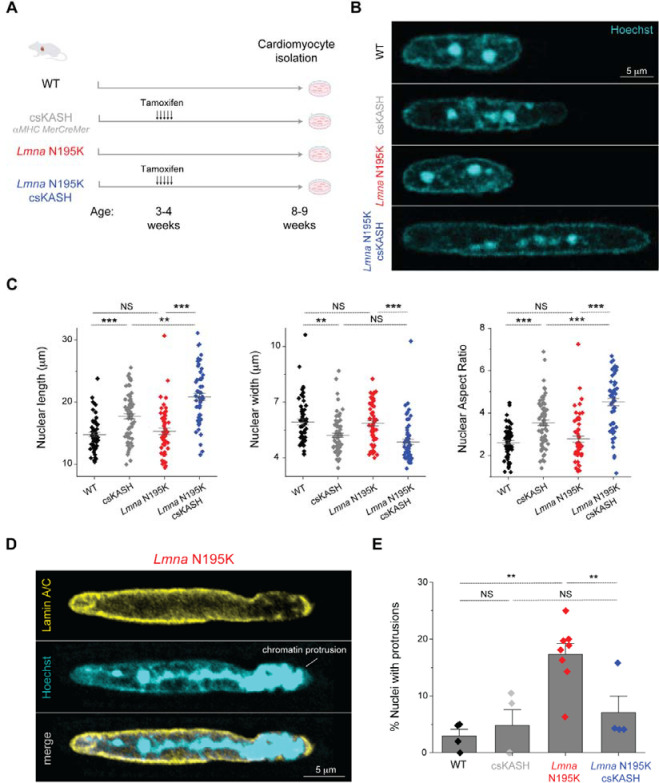
Cardiac specific LINC complex disruption protects against nuclear ruptures in *Lmna* N195K laminopathy. (A) Schematic presentation of the mouse models used in this study, timeline for tamoxifen / vehicle injections to induce cardiac specific LINC complex disruption, and end points for cardiomyocyte isolation. (B) Representative images of cardiomyocyte nuclear morphology at 8–9 weeks of age for the studied groups. (C) Quantification of nuclear length, width, and aspect ratio. WT: *N* = 4, *n* = 58. csKASH: *N* = 4, *n* = 69. *Lmna* N195K: *N* = 4, *n* = 55. *Lmna* N195K csKASH: *N* = 4, *n* = 59. (D) Nuclear rupture in *Lmna* N195K cardiomyocytes manifested by chromatin protrusion from the nucleus, with partial lamina coverage. (E) Quantification of % nuclei with protrusions, per animal for the indicated groups. WT: *N* = 4, *n* = 201. csKASH: *N* = 4, *n* = 129. *Lmna* N195K: *N* = 8, *n* = 403. *Lmna* N195K csKASH: *N* = 4, *n* = 284. Data presented as mean ± SE. Statistical significance determined by 1-way ANOVA with Bonferroni correction (*, *p* < 0.05; **, *p* < 0.01; ***, *p* < 0.001).

We next sought to investigate the role of specific cytoskeletal forces in nuclear damage driven by *LMNA* mutations. We utilized the previously described *Lmna*^N195K/N195K^ (hereafter ‘*Lmna* N195K’) mouse model that leads to heart failure and death within 12 weeks of age ^7^. Increased mechanical fragility of the nucleus is a hallmark of the *Lmna* N195K mutation, with a loss in nuclear stability approaching that observed with complete deletion of lamin A/C ^8,9^. Importantly, it has been recently demonstrated that cytoskeletal-nucleoskeletal decoupling via LINC complex disruption improves cardiac function and prolongs lifespan in *Lmna* N195K and other *Lmna* mutant mice ^14,15^. To probe the cardiomyocyte-specific effects of LINC disruption, we used the previously described inducible α*MHC MerCreMer* DN-KASH mouse model ^17^ (referred to as cardiac-specific KASH, or csKASH) and crossed it to the *Lmna* N195K mouse to generate a mouse model capable of inducible, cardiac specific LINC complex disruption. Fig. 3A summarizes the four mouse models and the corresponding experimental timelines used in this work. LINC complex disruption in the WT and *Lmna* N195K mice was induced by daily tamoxifen injections for 5 consecutive days at 3–4 weeks of age, allowing 5 weeks of LINC complex disruption in vivo before cardiomyocyte isolation at 8–9 weeks of age. Tamoxifen injection led to a DN-KASH-GFP ring that surrounded the nucleus in >95% of cardiomyocytes (*N* = 2, *n* = 24/25 cardiomyocytes), consistent with previous estimates of induction efficiency with this model ^17^. We confirmed LINC complex disruption upon induction of csKASH by the loss of perinuclear nesprin-1 in cardiomyocytes from both the WT and *Lmna* N195K backgrounds (Supplementary Fig. S2A).

We did not observe significant changes in nuclear dimensions between WT and *Lmna* N195K cardiomyocytes (Fig. 3B–C). LINC complex disruption (csKASH) resulted in elongated cardiomyocyte nuclei, and even longer nuclei with LINC complex disruption in the *Lmna* N195K model (*Lmna* N195K csKASH) (Fig. 3B–C). Nuclear elongation occurred independent of any corresponding changes in cellular length, width or aspect ratio (Supplementary Fig. S2B–C), instead indicating an altered balance between cytoskeletal forces acting on the nucleus and internal nuclear resistance, consistent with our previous work ^3^. Intriguingly, the increase in nuclear aspect ratio due to in vivo LINC complex disruption mirrored the alterations in nuclear morphology upon colchicine treatment (Fig. 2D, Fig. 3C), suggesting it may arise from a reduction in MT compressive forces.

While *Lmna* N195K nuclei did not change in dimensions compared to WT nuclei, we observed increased nuclear fragility and rupture in the form of chromatin that appeared to spill out of the nuclear membrane with the nuclear lamina only partially enclosing the protrusion (Fig. 3D). In *Lmna* N195K cardiomyocytes, 18% of the nuclei had blebs and chromatin protrusions (Fig. 3E). Importantly, in vivo LINC complex disruption rescued the *Lmna* N195K nuclear ruptures back to control levels (Fig. 3E). Of note, every observed chromatin protrusion (n=96) was located at the tips of the elongated nuclei, indicating a marked increase in susceptibility in this specific region, in agreement with a recent report on localized nuclear ruptures in cardiac specific *Lmna* KO mice ^11^. Taken together, these findings demonstrate that 5 weeks of LINC complex disruption in *Lmna* N195K cardiomyocytes protects from nuclear damage, concomitant with a marked increase in nuclear aspect ratio.

### In-vivo LINC complex disruption does not reduce nuclear strain during myocyte contraction

**Fig. 4:**
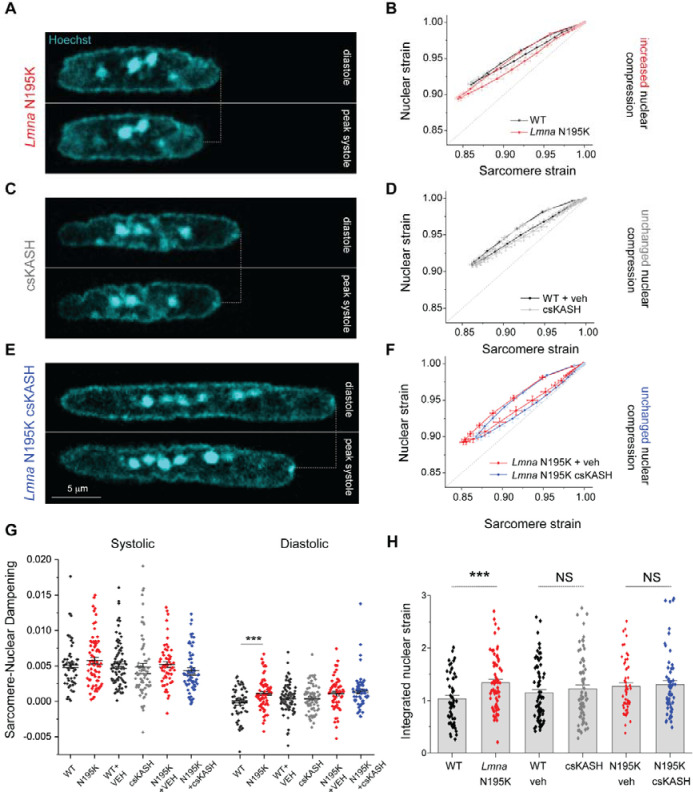
Increased active nuclear strain in laminopathy is not restored by cardiac specific in vivo LINC complex disruption. (A) Representative snapshots of Lmna N195K cardiomyocyte nuclei during diastole and peak systole. (B) Increased nuclear compression as evidenced by a mild downward shift in the laminopathy strain coupling curve. (C) Representative snapshots of cardiac specific LINC complex disruption in WT cardiomyocyte nuclei during diastole and peak systole with (D) no change in active strain coupling. (E) Representative snapshots of cardiac specific LINC complex disruption in Lmna N195K cardiomyocyte nuclei during diastole and peak systole with (F) no change in active strain coupling. (G) Quantification of sarcomere-nuclear strain dampening during the systolic and diastolic phases, for the indicated groups (see schematics in Fig. 1G). (H) Integrated nuclear strain over time during the contractile cycle for the indicated groups. WT: *N* = 4, *n* = 58. *Lmna* N195K: *N* = 4, *n* = 69. WT veh: *N* = 4, *n* = 81. csKASH: *N* = 4, *n* = 69. *Lmna* N195K veh: *N* = 4, *n* = 55. *Lmna* N195K csKASH: *N* = 4, *n* = 59. Data presented as mean ± SE. Statistical significance determined by two-tailed t-test (*, *p* < 0.05; **, *p* < 0.01; ***, *p* < 0.001).

A prevailing hypothesis for nuclear ruptures in laminopathies is that nuclei with a weakened lamina are more susceptible to mechanical damage induced by forceful sarcomeric contractions ^12^. Therefore, decoupling the nucleus from the cytoskeleton would prevent contractility-induced nuclear damage ^14^. To probe this hypothesis, we first asked whether *Lmna* N195K cardiomyocyte nuclei indeed undergo more strain during the contractile cycle. Representative images of *Lmna* N195K nuclei at diastole and peak systole are depicted in Fig. 4A. Consistent with a more deformable nucleus ^8^, *Lmna* N195K cardiomyocytes showed increased nuclear compression during sarcomere contraction (Supplementary Fig. S3A) and nuclear re-lengthening that lagged behind sarcomere re-lengthening. This resulted in a downward shift in the strain coupling curve (Fig. 4B), increased diastolic sarcomere-nuclear dampening (Fig. 4G), and a mild but significant increase in the integrated nuclear strain over the contractile cycle in *Lmna* N195K myocytes (Fig. 4H). This provides the first direct evidence for increased nuclear strain upon contraction of mature mutant *Lmna* cardiomyocytes.

We next hypothesized that LINC complex disruption may rescue the increased active strain experienced by *Lmna* N195K nuclei. Representative images of nuclei from csKASH and *Lmna* N195K csKASH mice at diastole and peak systole are depicted in Fig. 4C and 4E respectively. Surprisingly, there was no reduction in nuclear compression following cardiac specific in vivo LINC complex disruption in csKASH cardiomyocytes (Supplementary Fig. S3B), and therefore no change in the overall strain coupling curve (Fig. 4D) nor sarcomere-nuclear dampening (Fig. 4G). Taken together with our in vitro AdV DN-KASH results (Fig. 2F), this data indicates that an intact LINC complex is not required for transferring the majority of sarcomeric strain into the nucleus during myocyte contraction. Consistently, in *Lmna* N195K csKASH myocytes neither nuclear compression during contraction (Supplementary Fig. S3C), sarcomere-nuclear dampening (Fig. 4G), nor active strain coupling (Fig. 4F) were altered compared to *Lmna* N195K mutant littermates injected with vehicle control. Quantification of the integrated nuclear strain during the contractile cycle revealed that the increased nuclear strain in *Lmna* N195K mutants was not rescued by in vivo LINC complex disruption (Fig.4H). Together, these results indicate that while *Lmna* N195K mutant nuclei experience increased strain over the myocyte contractile cycle, this effect is not rescued by LINC disruption. This argues against the hypothesis that LINC complex disruption confers cardioprotection by reducing active nuclear strain.

### In-vivo LINC disruption eliminates the perinuclear microtubule cage.

**Fig. 5:**
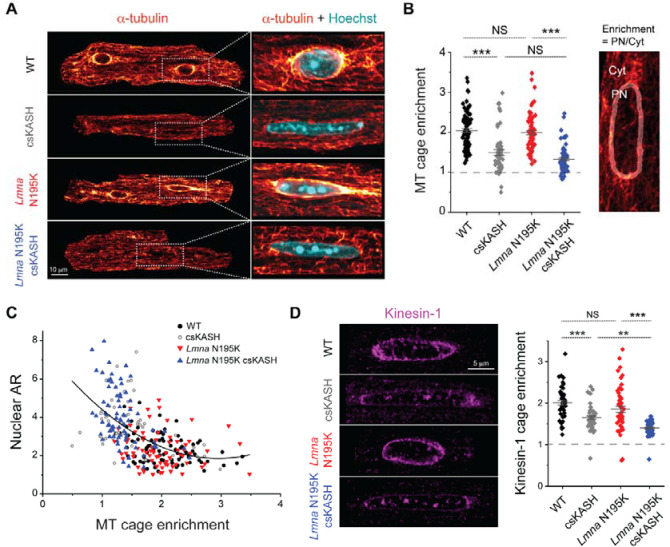
The nuclear effects caused by in vivo LINC complex disruption are dominated by a decoupled MT network. (A) Representative mid-plane immunofluorescence images of the MT network (α-tubulin), with a zoom in on a merge with labeled nuclei (Hoechst) in the WT, csKASH, *Lmna* N195K*,* and *Lmna* N195K csKASH adult mouse cardiomyocytes. (B) Quantification of perinuclear MT enrichment defined as perinuclear (PN) to cytoplasmic (Cyt) α-tubulin ratio (illustrated on the image on the right). WT: *N* = 3, *n* = 85. csKASH: *N* = 2, *n* = 51. *Lmna* N195K: *N* = 3, *n* = 70. *Lmna* N195K csKASH: *N* = 3, *n* = 75 (C) Nuclear aspect ratio (AR) as a function of perinuclear MT enrichment. Black line is 2^nd^ order polynomial fit, R^2^ = 0.326. (D) Representative mid-plane immunofluorescence images of kinesin-1 and quantification of perinuclear kinesin-1 enrichment*,* defined as PN to Cyt kinesin-1 ratio. WT: *N* = 2, *n* = 46. csKASH: *N* = 2, *n* = 51. *Lmna* N195K: *N* = 2, *n* = 50. *Lmna* N195K csKASH: *N* = 2, *n* = 59. Data presented as mean ± SE. Statistical significance determined by one-way ANOVA with Bonferroni correction (*, *p* < 0.05; **, *p* < 0.01; ***, *p* < 0.001).

Given that in vivo LINC complex disruption protected *Lmna* N195K nuclei from chromatin protrusions without decreasing the strain on the nucleus during contraction, we next explored other potential sources of mechanical stress on the nucleus. We hypothesized that nuclear elongation upon in vivo LINC complex disruption may be a consequence of reduced MT compressive forces on the nucleus. Consistently we observed almost complete elimination of the MT cage (measured as perinuclear to cytosolic α-tubulin enrichment) following cardiac specific in-vivo LINC complex disruption in WT and *Lmna* N195K cardiomyocytes (Fig. 5A–B). The dense perinuclear MT cage in primary cardiomyocytes was specifically enriched at the longitudinal nuclear tips (Supplementary Fig. S4A), which might contribute to increased mechanical forces at this location. In agreement, all chromatin protrusions in laminopathy cardiomyocytes were observed at the nuclear tips.

We leveraged intracellular heterogeneity to check if nuclear aspect ratio is dependent on the levels of perinuclear MT enrichment. Our data demonstrated a biphasic relationship between nuclear aspect ratio and the perinuclear MT cage (Fig. 5C, fitted with 2^nd^ order polynomial, R^2^ = 0.326). In all groups, we found a constant nuclear aspect ratio for MT cage enrichment ≥ 2. However, for lower levels of perinuclear MT enrichment a steep increase in the nuclear aspect ratio is observed, and the longest nuclei, where MT enrichment approaches 1 (no perinuclear to cytosolic α-tubulin enrichment), belong to the LINC complex disrupted *Lmna* N195K cardiomyocytes (blue). Together, these observations suggest that the nuclear elongation caused by in-vivo LINC complex disruption in adult cardiomyocytes is likely driven by decoupling of the MT network from the nucleus, with even steeper elongation in lamin mutant nuclei likely due to their increased deformability.

MTs interact with the LINC complex via kinesin motors, and nesprins are required to recruit kinesin motors and MTs to the nuclear periphery. In agreement with earlier reports, ^8,15,20^, we found that kinesin-1 is depleted from the perinuclear space in LINC complex disrupted WT and *Lmna* N195K cardiomyocytes (Fig. 5D). Consistently, we also observed robust perinuclear kinesin-1 depletion with partial loss of the perinuclear MT cage following 48 h AdV DN-KASH transduction in vitro in adult rat cardiomyocytes (Supplementary Fig. S4B–C). These findings suggest that LINC complex disruption prevents the attachment of nesprins to the perinucleus, which leads to rapid loss of kinesin motors and eventual loss of the perinuclear MT cage. Furthermore, both the in-vitro and in-vivo LINC complex disruption models suggest that nesprins recruit kinesin motors and MTs to the nuclear periphery.

### MT disruption protects *Lmna* mutant cardiomyocytes from nuclear damage.

**Fig. 6:**
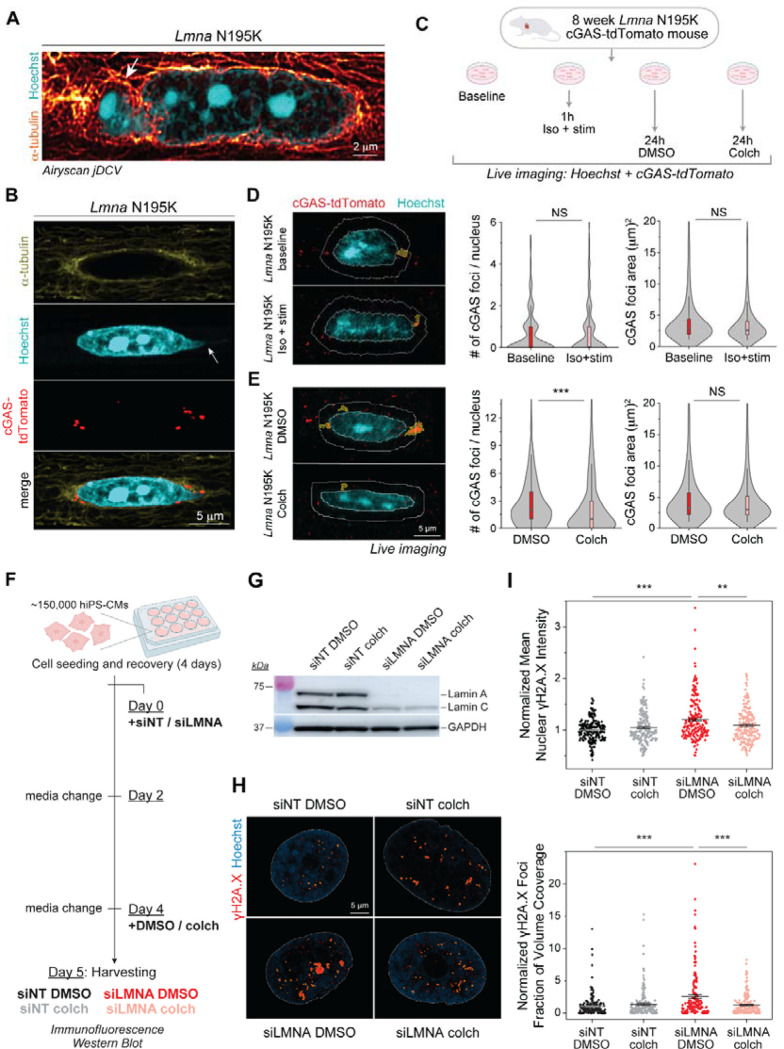
MT disruption protects from nuclear damage in *Lmna* N195K and hiPSC-cardiomyocytes. (A) Super resolution airyscan jDCV mid plane image of a *Lmna* N195K cardiomyocyte nucleus (Hoechst) with the dense MT network (_α_-tubulin) penetrating the chromatin protrusion. (B) Immunofluorescence mid plane image of the dense MT network (_α_-tubulin) on the tip of *Lmna* N195K nucleus, associated with a chromatin protrusion (Hoechst, arrow), and cGAS foci (cGAS-tdTomato) indicative of nuclear envelope ruptures. (C) Experimental design for live imaging of perinuclear cGAS-tdTomato foci in a *Lmna* N195K mouse model, following in-vitro stimulation in the presence of isoproterenol or colchicine treatment. Representative maximum intensity projection images of nuclei (Hoechst) and perinuclear cGAS-tdTomato foci, from (D) freshly isolated (baseline), and contractility induced (Iso + stim) cardiomyocytes, and (E) DMSO or colchicine treated cardiomyocytes. 2 μm perinuclear rings used for foci identification are indicated with white lines, and the detected cGAS foci are highlighted in yellow (left). Quantification of the number and area of perinuclear cGAS foci, per nucleus (right). *N* = 2, *n* = 1060 (baseline), *n* = 780 (1 h iso + stim), *n* = 961 (24 h DMSO), *n* = 1141 (24 h colch). Boxplots represent the mean ± 25–75^th^ data percentile. Statistical significance determined by two-tailed t-test. (F) Experimental design for the examination of DNA damage in hiPSC-derived cardiomyocytes upon transfection with non-targeted siRNA (siNT) or *LMNA-*targeted siRNA (siLMNA), and subsequent DMSO or colchicine (colch) treatment. (G) Western blots from replicates 2/3 (whole-cell lysates were pooled together) showing the siRNA-mediated lamin A/C knock-down in hiPSC-derived cardiomyocytes. GAPDH was used as a loading control. (H) Quantification of mean nuclear γH2A.X intensity (top) and γH2A.X foci fraction of nuclear volume coverage (bottom). Each replicate is normalized to the mean value of the NT DMSO group. (I) Representative mid-plane images of nuclei (Hoechst) and γH2A.X foci, from the four experimental groups of hiPSC-derived cardiomyocytes. Hoechst was used to outline the nuclei (white line), and the detected γH2AX foci are highlighted in yellow (left). Data presented as mean ± SE. *N* = 3, *n* = 199 (NT DMSO), *N* = 3, *n* = 197 (NT colch), *N* = 3, *n* = 192 (si*LMNA* DMSO), *N* = 3, *n* = 203 (*siLMNA* colch). Statistical significance determined by one-way ANOVA with Bonferroni correction (*, *p* < 0.05; **, *p* <0.01; ***, *p* < 0.001).

Our data suggests that nuclear damage in the *Lmna* N195K cardiomyocytes may be driven by the dense perinuclear MT cage that exerts LINC-dependent compressive forces on the compromised nuclear lamina. In support, super-resolution images of the MT network in *Lmna* N195K cardiomyocytes showed penetration of MTs into the protruded chromatin at the nuclear tip (Fig. 6A). To directly test the role of MTs in nuclear ruptures, we used transgenic mice expressing a fluorescent cGAS-tdTomato reporter line ^8^ crossed with the *Lmna* N195K mouse model to allow quantification of nuclear rupture sites in live cardiomyocytes. Live imaging of freshly isolated cardiomyocytes revealed cGAS-tdTomato foci in and around the nucleus in the *Lmna* N195K group that were significantly increased compared to cGAS-tdTomato WT mice (Supplementary Fig. S5A–B). Overall, 57% of *Lmna* N195K myocytes demonstrated cGAS foci at the nuclear periphery, relative to 18% of WT mice. In *Lmna* N195K myocytes, cGAS foci were mostly observed at the nuclear tips where MTs were enriched, at sites with or without chromatin protrusions (Fig. 6B).

We first asked whether augmented systolic workload would lead to additional nuclear ruptures in cardiomyocytes isolated from cGAS-tdTomato *Lmna* N195K mice. We electrically stimulated cardiomyocytes for 1 hour at 0.5 Hz in the presence of isoproterenol to increase contractile workload and compared cGAS foci to non-stimulated cells (Fig. 6C). While cGAS signal could be detected in the cytosol (Supplementary Fig. S5D), we restricted our analysis to perinuclear ruptures within 2 μm of the nuclear border. The perinuclear rings for foci detection are marked in white, and the detected cGAS foci are labeled in yellow in Fig. 6D–E. Induced contractility by electrical stimulation in the presence of isoproterenol did not affect the number of perinuclear cGAS foci, nor the size of the foci (Fig. 6D right). This data further indicates that sarcomere contractility is not the primary driver of nuclear damage in laminopathy.

We next tested the alternative hypothesis that MT compressive forces drive nuclear damage. We disrupted the MT network in quiescent cardiomyocytes in vitro with 1 μM colchicine for 24 h and compared cGAS-tdTomato foci to cells treated for 24 h with DMSO (Fig. 6C). With respect to nuclear morphology, colchicine treatment induced nuclear elongation as expected (Supplementary Fig. S5C). MT disruption resulted in a decrease in the number of perinuclear cGAS-tdTomato foci, with no change in their size (Fig. 6E right). This data indicates that 24h MT disruption prevented the formation of new nuclear envelope ruptures in *Lmna* N195K cardiomyocytes, without modulating the size of pre-existing ruptures.

We further probed the role of MT compressive forces in a complementary model of *LMNA* deficient, human induced pluripotent stem cell-derived cardiomyocytes (hiPSC-CMs), which exhibit mechanically induced DNA damage ^8,10,12,21,22^. We transfected hiPSC-CMs with *LMNA*-targeted siRNA (siLMNA) or non-targeted siRNA as controls (siNT) for 5 days and disrupted MTs with colchicine on the last day before cell harvesting (see experimental design in Fig. 6F). Lamin A/C knock-down was confirmed by western blot analysis (Fig. 6G and Supplementary Fig. S5E) and DNA damage was assessed by immunofluorescence for γH2A.X, an early marker for double-stranded DNA breaks ^23^. Fig. 6H depicts representative nuclei from hiPSC-CMs, demonstrating increased DNA damage in the *LMNA* depleted nuclei that is restored upon MT disruption. To quantify DNA damage, we considered both γH2A.X signal intensity and foci size. Both metrics of mean nuclear γH2A.X intensity (Fig. 6I top), and γH2A.X foci fraction of volume coverage (Fig. 6I bottom) show significantly increased DNA damage upon *LMNA* depletion that is rescued by colchicine treatment. Similar findings for the number and volume of γH2A.X foci are shown in Supplementary Fig. S5F–G. Taken together, our findings demonstrate that reducing MT compressive forces in lamin A/C deficient cardiomyocytes protects from nuclear damage in the form of chromatin protrusions and DNA damage responses.

### Computational model of nuclear damage due to resting cytoskeletal forces.

**Fig. 7:**
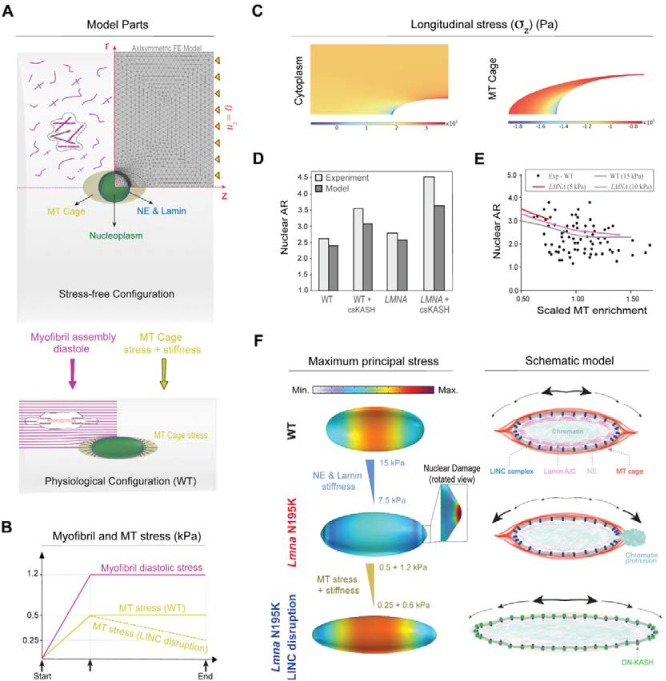
A computational model to explain nuclear damage in resting *LMNA* mutated cardiomyocytes and its rescue through LINC complex disruption. (A) Axisymmetric finite element (FE) model considering an imaginary stress-free configuration (top) for cardiomyocytes consisting of a round nucleus (which is further divided into nucleoplasm, and nuclear envelope and its underlying lamina), an ellipsoid MT cage, and the surrounding randomly distributed unassembled myofibrils. Diastolic contractility, geometric constraints of the myocardium, restoring forces of titin proteins, and compressive MT forces work in concert to deform this initial configuration to the physiological stressed configuration (bottom) where the nucleus is elongated, and the myofibrils are aligned. (B) Variations of the myofibril diastolic stress and MT compressive stress over the simulation time. (C) Distribution of the longitudinal stress component in the cytoplasm and the MT cage in physiological conditions. (D) Comparison between the model predictions and the experimental data for nuclear aspect ratio for our four different groups. (E) Simulation results for variation of the nuclear aspect ratio as a function of (scaled) MT enrichment for WT and *LMNA* mutated groups. (F) Simulations show that beyond a critical value for the MT compressive stresses, a form of instability emerges at the nuclear tips in which the location of maximum principal stress shifts from the middle of the nucleus to the tips (left, top and middle). Continuing the simulation after the instability by reducing MT compressive forces (simulating LINC complex disruption) shows that the nucleus becomes thinner and longer, with the maximum principal stress returning to the middle part (left, bottom). These observations are schematically shown in the right panel.

To gain a better understanding of the underlying forces that dictate cardiomyocyte nuclear morphology, nuclear ruptures upon lamina compromise, and the protective effect of LINC complex disruption, we developed a computational finite element (FE) axisymmetric model for the resting cardiomyocyte (Fig. 7A). In this model, we explicitly consider the myocyte nucleus, its surrounding MT cage, and the myofibrils (cytoplasm) as components involved in nuclear deformations. The nucleus is further divided into two parts: 1) nucleoplasm, and 2) the nuclear envelope and its underlying lamina. Experimental observations suggest that there is a complex prestress field in resting mature cardiomyocytes ^3,24,25^. This field results from resting cytoskeletal forces and plays a crucial role in governing the nuclear shape and stiffness in these rod-shaped cells ^3,24^. To induce this prestress field in our model, we first consider a (imaginary) cylindrical stress-free configuration for the cardiomyocytes where the nucleus is round, and the sarcomere units are not assembled yet (Fig. 7A, Supplementary Fig. S6 and methods for more details). We further restrict axial displacement $\left(u_{z}=0\right)$ of the cell ends (Fig. 7A) to mimic the geometric constraints imposed by the myocardial microenvironment ^25^ and the restoring stresses induced by titin springs ^26^. Furthermore, we add an isotropic and homogenous compressive stress field with magnitude $\sigma_{MT}$ to the perinuclear MT cage to capture the pushing forces mediated by MT polymerization ^3^ and MT motors (such as kinesin-1) on the nucleus. We assume that in the stress-free configuration, actomyosin fibers are distributed randomly and thus their associated contractility (shown by tensor $\rho_{ij}$ in our model) is initially isotropic and uniform everywhere in the cytoplasm with magnitude $\rho_{0}$. Note that $\rho_{ij}$ that shows the cardiomyocyte contractility at rest (diastole) ^26^, which differs from its active contractility during systole.

We next increase $\rho_{0}$ and $\sigma_{MT}$ from zero (stress-free configuration) to reach the physiologically stressed (WT) configuration (Fig. 7B). As a result, the cytoplasm contracts in the radial direction while its length remains constant (Supplementary movie 3), leading to the development of an anisotropic stress field within the cell. This stress field triggers stress-activated signaling pathways which result in polarization of actomyosin fibers ^27,28^ and assembly of myofibrils in the direction of maximum principal stress ^29^. Our simulations show that this principal direction is perfectly aligned with the direction of myofibrils in the cardiomyocytes (Fig. 7A bottom and Supplementary Fig. S6B). Furthermore, our simulations show that myofibril maturation imposes significant lateral compressive forces on the nucleus, leading to its elongation in the axial direction (Fig. 7A bottom). Nuclear elongation is however resisted by elastic compression of surrounding MTs and their pushing forces $\left(\sigma_{MT}\right)$ Together, these interactions induce the prestress field in the resting cardiomyocyte (Supplementary Fig. S6A). Our model predicts that maximum and minimum myofibril tensions happen at the short and long tips of the MT cage, respectively, while far from this cage, tension is nearly constant (Fig. 7C). In contrast, maximum and minimum axial MT compression happens at the long and short tips of the nucleus, respectively. Notably, the obtained values for diastolic tension of myofibrils (2-3kPa) and axial compression of MTs (1-1.8kPa) are in a good agreement with their corresponding experimental estimations ^24–26^.

We next explore model predictions for nuclear morphology changes due to lamin mutations and LINC complex disruption. To simulate laminopathy, we reduce the stiffness of the nuclear envelope and its underlying lamina network ^30,31^. To simulate LINC complex disruption, we decrease both the stress (Fig. 7B) and the stiffness of the MT cage (Fig. 7F), as supported by our observation that perinuclear kinesin-1 (Fig. 5D) and microtubule enrichment (Fig. 5B) are lost due to LINC complex disruption. Our simulations reveal that LINC complex disruption increases the nuclear aspect ratio, which is further exacerbated upon *LMNA* depletion, closely matching our experimental findings (Fig. 7D). This result highlights the significant role of MT enrichment around the nucleus in shaping its morphology. To examine this role in more detail, we then vary MT enrichment in our model by changing the cage stress and stiffness (Supplementary Fig. S6D and methods). Our simulations reproduce the experimentally observed biphasic relationship between nuclear aspect ratio and MT enrichment in the perinuclear region (Fig. 7E).

Our simulations also show that for any specified stiffness of the nuclear envelope and its underlying lamina, there exists a critical value for the MT enrichment which, beyond that, a form of instability emerges at the nuclear tips (Fig. 7E–F). Interestingly, the appearance of this instability is concomitant with the shift of the location of maximum principal stress (tension) from the middle of the nucleus to its long tips (Fig. 7F). Recent experimental studies ^32^ indicate that the nuclear lamina becomes diluted at the long tips, where the Gaussian curvature is high. Consequently, the combination of high tension and low lamina concentration makes the nuclear tips the most likely places to form nuclear lamina gaps, ultimately leading to nuclear damage and rupture ^33,34^. This model prediction is also consistent with our experimental observation that nuclear ruptures are always observed at the tips. Together, these simulation results suggest that compressive forces of the MT cage in the resting cardiomyocyte are sufficient to drive nuclear damage in *LMNA*-mutated cardiomyocytes.

Finally, we aimed to simulate rescue of the nuclear envelope rupture in *LMNA*-mutated cardiomyocytes through LINC complex disruption. After appearance of the instability at the nuclear tips, we simulate LINC complex disruption by decreasing stress and stiffness of the MT cage (Fig. 7B). As a result, the instability disappears, and the nucleus becomes thinner and longer while the location of the maximum principal stress comes back to the middle of the nucleus (Fig. 7F). This result provides a quantitative explanation for rescue of nuclear damage by LINC complex disruption in *LMNA*-deficient cardiomyocytes.

Overall, our experimental and modeling data suggest that the elongation of the nucleus in the mature cardiomyocyte is regulated by the balance between cross-sectional myofibril compression and the perinuclear MT cage that provides axial compressive forces to resist elongation (Fig. 7F, right). A compromised lamina leads to redistribution of the maximum principal stress to the nuclear tips, which contributes to nuclear envelope rupture and chromatin protrusions. Decoupling the nucleus from MT-based forces, via LINC complex disruption or direct MT disruption, redistributes the stress away from the tips, thus protecting the nucleus from new ruptures and allowing repair of the existing damage.

## Discussion

This study investigates force transmission into the cardiomyocyte nucleus during contraction and at rest to elucidate how MT compressive forces drive nuclear damage in laminopathy. We describe a novel method to measure active strain transfer from the contracting sarcomeres into the nucleus, and further demonstrate that sarcomere contractility is not a primary driver of nuclear ruptures in *Lmna*-DCM. Alternatively, we show that nuclear morphology and nuclear ruptures in *Lmna* mutants depend upon a dense cage of perinuclear MTs and associated kinesin motors. Either LINC complex disruption or direct MT depolymerization eliminates MT compressive forces and protects from nuclear ruptures and DNA damage. We further develop a computational model that demonstrates redistribution of stresses to the longitudinal tips of *LMNA* deficient nuclei, and restoration of stress distribution upon reduction in MT cage stress and stiffness. Therefore, our data support a model where local MT compressive forces, but not sarcomere contractility, induce damage at sites of nuclear fragility in laminopathic cardiomyocytes. Protection from nuclear ruptures can thus be achieved by disruption of MT-LINC interactions.

*LMNA* mutations disproportionally affect mechanically active tissues such as the heart, and nuclear fragility is hypothesized to be the main pathogenic driver of *LMNA* associated DCM. Active actomyosin contractility and the MT network have been implicated in laminopathy phenotypes ^8,12,14,15^, but dissecting their contribution to nuclear damage in the mature cardiomyocyte was technically challenging. Several studies reported on nuclear strain in response to passive stretch in non-muscle cells ^1,35^, or spontaneously contracting non-mature cardiomyocytes ^24,36^ but provided inconsistent insights on the mechanical cytoskeletal-nucleoskeletal coupling. Our active strain coupling approach provides the first demonstration of sarcomere-nuclear strain coupling in electrically stimulated mature cardiomyocytes. We report physiologically relevant sarcomere strains of >10%, and high temporal resolution for instantaneous strain coupling with the nucleus, providing amplitude and kinetics for systolic and diastolic phases.

We demonstrate that the strain on the nucleus in the mature cardiomyocytes is tightly coupled to the sarcomere strain during stimulated contraction, with consistent dampening of the nuclear strain amplitude. Surprisingly, this active coupling is only mildly disturbed following perturbation of the non-sarcomeric cytoskeletal or nucleoskeletal mechanical properties (e.g., MT network, LINC complex, lamin A/C). This suggests that the central location of the nuclei in the cardiomyocyte facilitates nuclear deformation largely by the surrounding myofibrils that squeeze on the nuclei during contraction, independent of direct physical connection via LINC complexes. Yet acute disruption of the LINC complex or the MT network (adult rat cardiomyocytes) still reveals their distinct roles in active nuclear strain coupling. Depolymerization of the MTs specifically altered the nuclear relaxation kinetics during diastole, without changing nuclear strain amplitude. This effect is consistent with MT buckling during compression ^19^ providing restoring force to the nucleus during relaxation. In contrast, in-vitro LINC complex disruption reduced the overall strain amplitude on the nucleus, echoing previous reports in non-muscle cells or non-mature cardiomyocytes ^1,36^. We further show that when similar disruption of the LINC complex (by a dominant negative KASH construct) is performed for several weeks in vivo, we no longer observe reduction in the amplitude of the active nuclear strain. This discrepancy between the short term in vitro, versus longer-term in vivo LINC complex disruption effect on the active strain coupling is likely due to the adaptive remodeling of the cytoskeleton in the in vivo system, as also evident by the distinct alterations in nuclear morphology, and the extent of perinuclear MT cage elimination.

With respect to laminopathy and the protective mechanism of LINC complex disruption, our strain coupling analysis demonstrates a mild increase in the active nuclear strain in *Lmna* N195K cardiomyocytes. Such an increase in nuclear strain during each contractile cycle might point to a potential mechanism of rupture in the compromised nuclei. However, we demonstrate that cardiac specific LINC complex disruption protects *Lmna* N195K nuclei from ruptures independent of any restoration in active nuclear strain, suggesting that actomyosin contractility is not the dominant driver of nuclear damage in *Lmna* N195K cardiomyocytes. These findings are in agreement with the dispensability of nesprin-1’s actin binding domain on striated muscle structure and function in adult mice ^20^. In support, we further demonstrated that increased contractile load in vitro did not increase the rate of cGAS-tdTomato foci in laminopathy cardiomyocytes. However, these results are limited by the short stimulation time (1 hour), and further investigation utilizing chronic alteration in actomyosin contractility is required to conclude on its possible involvement in laminopathy associated nuclear ruptures.

Alternatively, our experimental and modeling results demonstrate that MT compressive forces at rest regulate nuclear morphology in the adult cardiomyocyte. During maturation cardiomyocytes undergo uniaxial elongation, and the centrally located nuclei are squeezed by the expending myofibrils, causing overall nuclear elongation ^25^. The dense perinuclear MT cage is also formed during cardiomyocyte maturation and provides balancing compressive forces to the nucleus, with a denser concentration at the myofibril void spaces along the longitudinal nuclear tips (Supplementary Fig. S1B). We demonstrate that elimination of the compressive MT forces from the nuclear tips results in nuclear elongation, and in the context of mutant *Lmna* the structurally compromised nuclei undergo even greater elongation upon MT cage elimination.

Importantly, we show that compressive MT forces that restrict nuclear elongation are responsible for the nuclear damage associated with laminopathy DCM. In agreement with recent findings, we show that LINC complex disruption in WT cardiomyocytes eliminates the MT cage ^15^. We further demonstrate the loss of the perinuclear MT cage as well as the interacting kinesin-1 motors upon cardiac specific LINC disruption in laminopathy, concomitant with a reduction in NE damage in the form of chromatin protrusions. A protective role of kinesin-1 depletion was also recently demonstrated in *Lmna* KO developing myotubes, where MT associated motors are required for nuclear migration during maturation ^8^. Our findings further support the role of MT compressive forces in *Lmna*-DCM associated nuclear damage by a reduction in new nuclear ruptures sites (utilizing the cGAS-tdTomato reporter) and reduced DNA damage (pinpointed by the γH2A.X marker) upon direct disruption of the MT network. In addition, our computational model incorporates tensile forces from maturing myofibrils, stiffness of the nuclear lamina, and combined stress and stiffness from the MT cage and associated motors. Model simulations demonstrate the redistribution of overall nuclear stress to the longitudinal nuclear tips in *Lmna* mutated nuclei with intact MT forces, localizing a site of nuclear vulnerability. The localized stress at the nuclear tip is restored to the center of the nucleus when both MT stiffness and stress are reduced, concomitant with nuclear elongation, in agreement with our experimental findings.

This study focused on the role of cytoskeletal forces as a pathological driver in *Lmna* related DCM. While the role of the nuclear lamina in chromatin organization and gene expression was outside the scope of this study, it is also hypothesized to participate in the molecular pathogenesis of laminopathies ^13,37,38^. The dual role of lamin A/C in providing nuclear integrity but also organizing chromatin at the nuclear periphery, which in turn affects nuclear mechanical stability, points to potentially integrated effects of structural stability and chromatin organization in driving *LMNA* related pathology ^39^.

In summary, our study introduces a novel method to investigate active sarcomere-nuclear strain coupling in the primary beating cardiomyocyte, and implicates compressive MT forces at rest, but not active sarcomere contractility, in *LMNA*-DCM associated nuclear damage. We conclude that targeting specific interactions between MTs and the LINC complex should be pursued as a potential strategy to confer cardioprotection in *LMNA*-DCM.

## Methods

### Animals.

Animal care and use procedures were performed in accordance with the standards set forth by the University of Pennsylvania Institutional Animal Care and Use Committee and the Guide for the Care and Use of Laboratory Animals published by the US National Institutes of Health. Protocols were approved by the University of Pennsylvania Institutional Animal Care and Use Committee. All animals provided by the Lammerding Lab at Cornell were bred and maintained according to relevant guidelines and ethical regulations approved by the Cornell University Institutional Animal Care and Use Committee, protocol 2011–0099. Both rats and mice were housed in a facility with 12-h light/dark cycles and provided ad libitum access to water and chow. Temperature and humidity were checked daily to ensure that these parameters stay within appropriate ranges (20–26 C, 30–70%, respectively). αMHC Cre, KASH (csKASH) and *Lmna*^N195K/N195K^ mice have been described previously ^7,17^ and were back-crossed at least seven generations into a C57BL/6 line ^8^. To generate αMHC Cre^+/−^ KASH^+/−^ *Lmna*^N195K/N195K^ mice (*Lmna* N195K csKASH), male αMHC Cre^+/−^ KASH^−/−^ *Lmna*^N195K/+^ and female αMHC Cre^−/−^ KASH^+/−^ *Lmna*^N195K/+^ mice were crossed to create experimental and control littermate mice. *Lmna* mutant mice were provided with gel diet supplement (Nutri-Gel Diet, BioServe) to improve hydration and overall improve health and quality of life. To induce cardiomyocyte specific KASH-mediated LINC complex disruption, 30mg/ml tamoxifen suspended in sunflower oil or vehicle was injected intraperitoneally 5x daily starting at approximately 3 weeks of age followed by a 1 week wash out. To generate *Lmna*^N195K/N195K^ mice expressing cGAS-tdTomato, the previously described cGAS/MB21D1-tdTom transgenic mouse ^8^ was crossed into the *Lmna N195K* background to generate 3×FLAG-cGAS^E225A/D227A^-tdTomato positive *Lmna*^N195K/N195K^ mice within two generations. Single cardiomyocyte data was collected at stated timepoints.

### Adult rat and mouse cardiomyocyte isolation and culture.

Primary adult ventricular myocytes were isolated from 8- to 12-week-old Sprague Dawley rats, or 8–9 week-old mice using Langendorff retrograde aortic perfusion with an enzymatic solution as previously described ^19^. Briefly, the heart was removed from an anesthetized rodent under isoflurane and retrograde-perfused on a Langendorff apparatus with a collagenase solution. The digested heart was then minced and triturated with glass pipettes to free individual cardiomyocytes. The resulting supernatant was separated and centrifuged at 300 rpm to isolate cardiomyocytes. These cardiomyocytes were then resuspended in cardiomyocyte media (Medium 199 (Thermo Fisher) supplemented with 1x insulin-transferrinselenium-X (Gibco), 1 μg/μL primocin (InvivoGen), and 20 mM HEPES, pH = 7.4 (UPenn Cell Center)) at low density, cultured at 37 C and 5% CO2 with the addition of 25 μmol/L of cytochalasin D in the media.

### Active sarcomere-nuclear strain coupling.

Active strain coupling was quantified by back-to-back measurement of sarcomere length contractility and nuclear deformation during electrically stimulated contractions in adult cardiomyocytes. Cardiomyocytes in culture media were loaded with 4μM Hoechst and transferred to a custom-fabricated cell chamber (IonOptix) mounted on an LSM Zeiss 880 inverted confocal microscope with 63×oil 1.4 numerical aperture objective. Experiments were conducted at room temperature, and field stimulation was provided at 1 Hz with a cell stimulator (MyoPacer, IonOptix). Rod shaped cells with stable contractions were selected, and baseline sarcomere length >1.7μm, and sarcomere length strain > 10% were used as inclusion criteria. For each cell, sarcomere length contractility was first measured with a transmitted light camera (IonOptix MyoCam-S) and real time optical Fourier transform analysis (IonWizard, IonOptix). For each cell, 5 steady state and consistent SL traces were recorded in a region of interest close to the nucleus, either above or below the nucleus. The microscope was then immediately switched to fast mode Airyscan confocal and Hoechst fluorescence was imaged with 405nm laser (lowest laser power), at 11 msec/frame (91 Hz), for 5 additional steady state contractions. Initial image analysis was performed using ZEN black software for Airyscan processing, which involves signal integration from the 32 separate sub-resolution detectors in the Airyscan detector and subsequent deconvolution of this integrated signal. Stimulation times were recorded with the sarcomere contractility and nuclear imaging files for offline alignment of the traces. An average sarcomere contractility trace was generated for each cell (IonWizard, IonOptix) and exported to Matlab (Mathworks R2022b) for further alignment with nuclear deformation traces. Nuclear image analysis was performed with Arivis V4D 4.0–4.1, by a pipeline to autosegment (Otsu) the nucleus object in each frame and export nuclear morphology parameters. Nuclear length and width traces were imported to Matlab, averaged for each cell, and aligned with the corresponding average sarcomere contractility trace (after interpolation to 91 Hz to match nuclear time trace). Sarcomere and nuclear strains were calculated by dividing the corresponding instantaneous strains by the baseline lengths prior to stimulation. For each experimental group 15–20 cardiomyocytes were recorded, from 3–4 biological replicates.

### hiPSC-derived cardiomyocytes:

Human induced pluripotent stem cell-derived cardiomyocytes (hiPS-CMs) were purchased from Ncardia (Nc-C-BRCM). Cells were maintained in RPMI supplemented with 10% FBS (Gibco, 16000044), 1% Penicillin-Streptomycin (Gibco, 15140122), and 2% B-27 (Gibco, 17504044). After 4 days in maintenance media, 150,000 cells were plated onto glass coverslips (for immunofluorescence) or into 12-well culture dishes coated with 10 μg/ml fibronectin (Sigma-Aldrich, F1141) diluted in DPBS with Mg^2+^ and Ca^2+^. Cells were then allowed to recover for 4 days in maintenance media. On day 0, cells were treated with siRNAs, against either a non-targeting control (siNT) or lamin A/C (siLMNA) at a concentration of 5nM using Lipofectamine RNAiMax transfection reagent diluted in Opti-MEM. Cells were incubated with siRNAs for 5 days total, and media was changed on days two and four. On day 4, cells were also treated with either DMSO as a control, or colchicine at a concentration of 1nM. 24 hours after DMSO or colchicine treatment, cells were harvested for downstream analysis.

### Immunofluorescence.

Primary rat and mouse cardiomyocytes were fixed in 4% PFA (Electron Microscopy Sciences) for 10 min, washed three times with PBS, and permeabilized in 0.1% TritonX-100 for 10 min at room temperature. After washing twice with PBS, cells were placed in blocking buffer (1:1 Seablock (Abcam) and 0.1% TritonX-100 (Bio-Rad) in PBS for at least 1 h at room temperature, then labeled with primary antibodies (see below) for 24–72 h at 4 °C. Cells were then washed three times in PBS, then labeled with secondary antibodies in PBS at room temperature for 2–4 h. Hoechst was added for the last 10 min of secondary incubation and cells were washed twice with PBS. Stained cells were mounted on #1.5 coverslips in Prolong Diamond Antifade Mountant (Thermo Fisher) for imaging. Slides were left to cure in the dark for at least 24 h prior to imaging. hiPS-CMs coverslips were fixed in 4% PFA for 10 minutes at room temperature, then rinsed in DPBS for 5 minutes three times. Cells were permeabilized with 0.5% TritonX-100 for 10 minutes, blocked in 1% BSA in PBS-T (8mM Na_2_HPO_4_, 150mM NaCl, 2mM KH_2_PO_4_, 3mM KCl, 0.05% Tween 20, pH 7.4) and incubated with primary and secondary antibodies diluted in PBS-T + 1% BSA for 1 hour each at room temperature. Samples were counterstained with DAPI solution (Sigma, D9542) for 10 minutes at room temperature, then rinsed with PBS and stored/imaged in SlowFade Gold Antifade Mountant (Invitrogen, S36936).

### Antibodies, labels, and pharmaceuticals.

Hoechst 33342 (Invitrogen, H3570); SPY650-DNA, SPY555-Tubulin, and SiR-actin (0.1μL/mL, Spirochrome); Anti-alpha Tubulin mouse monoclonal antibody, clone DM1A (1:500 abcam ab264493); Anti-KIF5B rabbit monoclonal antibody clone EPR10276(B) (1:500 abcam ab167429); Anti-Nesprin1 rabbit monoclonal antibody clone EPR14196 (1:250 abcam ab192234); Anti-Lamin A/C mouse monoclonal antibody clone 4C11 (1:500, Cell Signaling #4777); phospho-H2A.X (1:1000 05–636, Millipore Sigma). Goat anti-rabbit IgG AF 647 (1:1000, Life Technologies, A27040); Goat anti-mouse IgG AF488 (1:1000, Life Technologies, A11001).

Colchicine (1 μM in DMSO, Sigma-Aldrich), Isoproterenol (1 μM in DMSO), Blebbistatin (10 μM in DMSO, Cayman Chemical).

### Image acquisition.

*Live 3D primary cardiomyocytes:* For live 3D super-resolution imaging adult rat cardiomyocytes in culture media were loaded overnight with 0.1μL/mL SPY650-DNA (Spirochrome) to label nuclei, and 10μM Blebbistatin immediately before imaging, to prevent motion artifacts. Airyscan SR (super resolution) z-stacks were acquired with Zeiss 880 Airyscan confocal microscope with 63×oil 1.4 numerical aperture objective, and 640nm laser line in a glass bottom dish. Raw images were processed with a joint deconvolution plugin (ZEN 3.5 blue). *Live cGAS-tdTomato foci:* Live isolated cGAS-tdTomato cardiomyocytes were adhered to glass bottom dishes using MyoTak (IonOptix) and loaded with 4μM Hoechst and 10μM Blebbistatin immediately before imaging. Tile scan images were acquired at room temperature with Zeiss 980 Airyscan confocal microscope equipped with Plan-Apochromat ×20 air 0.8 numerical aperture objective. Two channels (tdTomato and Hoechst), 1 μm z-stacks tile scans were acquired, stitched and Airyscan processed using ZEN black software. Nuclei from dead or severely deformed cells, or with motion or stitching artifacts were excluded from analysis. *hiPSC-CM imaging*: Z-stacks (10 μm range with voxel size of 0.035 × 0.035 × 0.5 μm) of individual nuclei were acquired on a LSM Zeiss 980 Airyscan 2 confocal microscope. A Plan-Apochromat 63×oil 1.4 numerical aperture objective was used. Tile imaging was performed under 4x zoom and 2x bidirectional averaging. The SR-4Y Multiplexing (MP) acquisition mode was used for faster parallel pixel readout. Samples were excited with the 405 nm, 488 nm and 639 nm laser lines. For nuclei selection, the Hoechst channel was used to preview images and 33.7 × 33.7 μm regions of interest (ROI) were defined to frame individual nuclei. To ensure examination of properly individualized hiPSC-derived cardiomyocytes, ROIs containing more than one nucleus were not selected for imaging. Images were Airyscan processed using the ZEN black software.

### Image analysis.

Raw images were Airyscan processed with ZEN Black software and imported to Arivis V4D 4.0–4.1 for further analysis. Dedicated V4D analysis pipeline was generated to auto segment (otsu) the nuclei from 3D z-stacks or 2D maximum intensity projection (MIP) images, and export nuclear morphology parameters, such as nuclear volume, length, width, and aspect ratio. Perinuclear enrichment was calculated on MIP images from 3×1μm z-stacks slices (total 3μm) around the midplane of the nucleus. A perinuclear ring object was created from 0.5μm dilation of the nuclear object and subtraction of the nucleus. Nuclear pole enrichment was calculated from manually traced 2 μm wide objects for each of the long and short nuclear poles. cGAS foci were calculated from tile MIP images with auto segmentation (Otsu) of the nuclei from the Hoechst channel, and generation of 2μm perimeter perinuclear ring. Fixed intensity (>600AU, or >200AU for the WT vs *Lmna* N195K cGAS-tdTomato models) and size (>0.8μm^2^) thresholds for cGAS-tdTomato signal were used to segment the cGAS foci in the vicinity of the perinuclear ring. The segmented cGAS foci were used to quantify the percentage of cGAS positive myocytes. To prevent contamination by cytosolic cGAS intensity, only foci with >70% area within the 2μm diameter perinuclear rings were included as readouts for nuclear ruptures. hiPSC-CMs images were analyzed with custom Arivis V4D 4.1 pipeline to segment the Hoechst channel using the Otsu method to outline the nuclei in 3D z-stacks. γH2A.X foci were segmented using fixed intensity (>2000 A.U.) and volume (0.02–1000 μm^3^) thresholds. Nuclear volume and mean nuclear intensity of γH2A.X signal were calculated using the nuclear segmentation. Puncta volume, integrated volume of all intranuclear puncta, and number of puncta were calculated from the segmented γH2A.X images. The data were imported to Matlab (Mathworks R2022b) for pooling, and subsequent graphing and analysis was performed using Origin 2019 (OriginLab Corporation). The integrated volume of all γH2A.X foci within a nucleus was divided by the corresponding nuclear volume to calculate the fraction of the nuclear volume occupied by γH2A.X foci, or foci fraction of volume coverage. The integrated volume of all γH2A.X foci within a nucleus was divided by the number of foci in that nucleus to calculate the mean γH2A.X foci volume. Data points with a value greater than 3 standard deviations (SD) above the mean of the γH2A.X mean foci volume and/or of the number of γH2A.X foci were considered outliers and excluded from the analysis. These two parameters were used to identify outliers because they showed the highest variability among the measured parameters. Data were normalized to the mean of the NT DMSO group for each experimental replicate.

### Statistics.

Statistical analysis was performed using Matlab and OriginPro (Version 9 and 2018). Statistical test and information on biological and technical replicates can be found in the figure legends. For box plots, the mean line is shown, with whiskers denoting standard error (SE) from the mean. Statistical tests for each comparison are denoted in the figure legends.

### Computational model.

The adult cardiomyocyte is rod shaped and often binucleated, with cylindrical symmetry that is largely preserved with *Lmna* mutations and LINC complex perturbations. For simplicity, we leverage this symmetry and consider an axisymmetric model covering only a quarter of the cardiomyocyte (see Fig. 7A and Table S1). Using the COMSOL multi-physics software, we simulate nuclear morphological changes due to the mutation and LINC complex disruption. Fig. 7A illustrates a typical finite element mesh used for these simulations. According to this figure, the model comprises three main parts: 1) myofibrils (cytoplasm), 2) nucleus, and 3) perinuclear MT cage, which are explained in the following sections.

#### Myofibrils:

Adult cardiomyocytes are predominantly filled with myofibrils, encapsulated by the surrounding sarcolemma. Myofibrils are long contractile fibers that are comprised primarily of interdigitating actin and myosin filaments which are precisely held together by titin proteins ^40^. Experimental observations show that during diastole, actomyosin interaction is not fully off and thus there is an active contraction in resting cardiomyocytes ^26^. This contraction encounters resistance from restoring forces mediated by titin protein ^26^ and geometric constraints imposed by the myocardium microenvironment ^25^. Our simulations (discussed later) demonstrate that this diastolic contraction laterally compresses central nuclei, causing them to elongate in the axial direction. This nuclear elongation, in turn, compresses the surrounding MT cage, generating pushing elastic forces in the perinuclear MT cage. Consequently, a complex prestress field exists in the cardiomyocyte that governs the nuclear morphology changes. The existence of this resting stress field is supported by the observation that the elongated cardiomyocyte nuclei become round upon isolation ^24^. To induce this stress field in our model, we note that available experiments ^41^ show that myofibrils tend to assemble in the direction of maximum principal stress ^29^. This implies that the myofibrillar organization during maturation is mainly controlled by stress-activated signaling pathways (like calcium pathway) ^25^. Therefore, we employ our previously developed chemo-mechanical model ^28^ to capture this stress dependent assembly of the myofibrils. To this end, we first hypothesize an (imaginary) stress-free configuration for the cardiomyocyte, wherein the nuclei are round, and actomyosin fibers (myofibrils) are randomly distributed and not yet assembled (Fig. 7A). We further assume the following relation between the myofibrillar prestress field $\left(\sigma_{ij}^{mf}\right)$, the rest contractility (shown by tensor $\rho_{ij}$), and the corresponding strain field $\left(\varepsilon_{ij}\right)$ (see ^28^ for more details):

(S1) $$\sigma_{ij}^{mf}=\overset{‾}{K}\varepsilon_{kk}\delta_{ij}+2\overset{‾}{\mu }\left(\varepsilon_{ij}-\frac{1}{3}\varepsilon_{kk}\delta_{ij}\right)+{\overset{‾}{\rho }}_{0}\delta_{ij},$$

(S2) $$\rho_{ij}={\overset{‾}{\rho }}_{0}\delta_{ij}+{\overset{‾}{K}}_{\rho }\varepsilon_{kk}\delta_{ij}+2{\overset{‾}{\mu }}_{\rho }\left(\varepsilon_{ij}-\frac{1}{3}\varepsilon_{kk}\delta_{ij}\right),$$

where, we have:

(S3) $${\overset{‾}{\rho }}_{0}=\frac{\beta \rho_{0}}{\beta \;-\;\alpha_{v}},3\overset{‾}{K}=\frac{3K\beta \;-\;1}{\beta \;-\;\alpha_{v}},2\overset{‾}{\mu }=\frac{2\mu \beta \;-\;1}{\beta \;-\;\alpha_{v}},3{\overset{‾}{K}}_{\rho }=\frac{3K\alpha_{v}\;-\;1}{\beta \;-\;\alpha_{v}},2{\overset{‾}{\mu }}_{\rho }=\frac{2\mu \alpha_{v}\;-\;1}{\beta \;-\;\alpha_{v}},$$

in which β is the chemical stiffness, $\alpha_{v}$ is the chemo-mechanical feedback parameter, and K=E/(3(1-2v)) and μ=E/(2(1+v)) are the bulk and shear moduli with E and v as the elastic modulus and Poisson’s ratio, respectively. Furthermore, $\rho_{0}$ is the initial contractility which is zero in the stress-free configuration. The selected values for these model parameters are given in Tables. S2 and S3.

By increasing the initial contractility (i.e., $\rho_{0}$) (Fig. 7B), we then simulate the assembly of myofibrils, resulting in a tendency for the cardiomyocyte to shrink. However, this cell shrinkage is resisted, primarily in the longitudinal direction, by titin proteins and the geometric constraints of the cardiomyocyte microenvironment ^42^, as mentioned earlier. Accordingly, we restrict the longitudinal displacement of the cell at its two ends. Therefore, radial contraction occurs in the cell while its length remains fixed (see Supplementary movie S3). This constrained contraction gives rise to the generation of an anisotropic stress field inside the cytoplasm, as illustrated in Fig. 7C and supplementary Fig. S6A. As a result of this anisotropic stress field, the contractility tensor $\rho_{ij}$ will be no longer isotropic (see Eq. S2) since stress activated signaling pathways promote formation of myofibrils in the direction of maximum principal stress ^27,29^. Supplementary Fig. S6B illustrates the obtained directions for the maximum principal stress (maximum tensile stress) field inside the cytoplasm. Notably, there is a great match between these directions and the directions of myofibrils in the physiological conditions.

#### Nucleus:

The nucleus is primarily composed of two key mechanical components: chromatin and the nuclear envelope (NE) with its underlying lamina. The nuclear envelope includes nuclear membranes, and nuclear pore complexes (NPCs), while chromatin serves as the primary building block of the nucleoplasm. Experimental observations ^43^ indicate that chromatin plays a crucial role in resisting small nuclear deformations, while NE and its underlying lamina predominate in scenarios involving large nuclear deformations. These experiments further suggest that the lamina network, especially lamins A and C, exhibits significant strain stiffening, whereas chromatin remains linear even at large deformations. Consequently, we model the nucleoplasm (chromatin) as a linear elastic material with a Young’s modulus of 150 Pa ^27^ and the NE and its underlying lamina as a neo-Hookean hyperelastic layer with an elastic modulus of 15 kPa ^44,45^. This hyperelastic material model captures the strain stiffening of the NE and its underlying lamina upon large deformation. Furthermore, our in vivo observations demonstrate that relative nuclear volume changes due to lamin mutation and LINC disruption are less than 10 % (Fig. S6C). Based on these findings, we further consider the nucleoplasm (chromatin) to be nearly incompressible with 0.49, while the NE and its underlying lamina are modeled as a fully incompressible hyperelastic layer, similar to the approach in ^44^.

#### Perinuclear MT cage:

In adult cardiomyocytes, microtubule organizing centers are predominantly associated with the NE ^46^. MTs anchor to the NE through the LINC complex, particularly nesprin-1 proteins and kinesin motors ^15^, forming a dense and active cage around the nucleus. Consequently, MTs can exert active pushing forces on the nucleus through their polymerization and recruitment of the kinesin motors. This is supported by our super-resolution images (Fig. 6A), which reveal a wavy form of MTs around the nucleus, indicating that these rod-shaped fibers are under compression. To account for these active forces and for simplicity, we introduce an isotropic and homogenous compressive stress field with magnitude $\sigma_{MT}$ to the perinuclear MT cage (Eq. S4). We increase this external stress from zero in the stress-free configuration to 500 Pa when inducing the prestress field under WT (physiological) conditions (Fig. 7B). Furthermore, considering the observed microtubule wavelengths, typically between 2–3 μm (Fig. 6A–B), the compressive force experienced by the MTs is estimated to be 100pN or more ^47^. This force is 10 times larger than the force of MT polymerization and 14–15 times larger than the force applied by kinesin motors ^48^. Consequently, we assume that MTs are also under passive elastic compression in adult cardiomyocytes. Accordingly, we model the MT cage as an (active) linear elastic ellipsoid in the stress-free configuration. The constitutive equation for this cage can be written as:

(S4) $$\sigma_{ij}^{MT}=K_{MT}\varepsilon_{kk}\delta_{ij}+2\mu_{MT}\left(\varepsilon_{ij}-\frac{1}{3}\varepsilon_{kk}\delta_{ij}\right)-\sigma_{MT}\delta_{ij},$$

where $K_{MT}$ and $\mu_{MT}$ are the bulk and shear moduli of the MT cage, respectively. Fig. 7C and Supplementary Fig. S6E show the obtained stress field in the cage. According to these figures, the MT cage imposes maximum compressions on the long and short tips of the nucleus in the longitudinal (z direction) and radial (r direction) directions, respectively. This is consistent with our current observations that nuclear ruptures mostly occur at the long tips and also with our recent experimental findings ^3^ that MTs indent into desmin knockdown nuclei mostly around the short sides and in the radial direction.

## Supplementary Material

Supplement 1

Supplement 2

Supplement 3

Supplement 4
