# Supplementary material for: Microtubule forces drive nuclear damage in LMNA cardiomyopathy
Source: bioRxiv. 2025 Jun 2:2024.02.10.579774. Preprint. [Version 5] doi: 10.1101/2024.02.10.579774 (PMC11212868; doi:10.1101/2024.02.10.579774)
Supplement: Supplement 4 [file NIHPP2024.02.10.579774v5-supplement-4.pdf]

bioRxiv preprint doi: https://doi.org/10.1101/2024.02.10.579774; this version posted June 2, 2025. The copyright holder for this preprint (which was not certified by peer review) is the author/funder, who has granted bioRxiv a license to display the preprint in perpetuity. It is made available under aCC-BY 4.0 International license.

Methods

Animals. Animal care and use procedures were performed in accordance with the standards set forth by the University of Pennsylvania Institutional Animal Care and Use Committee and the Guide for the Care and Use of Laboratory Animals published by the US National Institutes of Health. Protocols were approved by the University of Pennsylvania Institutional Animal Care and Use Committee. All animals provided by the Lammerding Lab at Cornell were bred and maintained according to relevant guidelines and ethical regulations approved by the

bioRxiv preprint doi: https://doi.org/10.1101/2024.02.10.579774; this version posted June 2, 2025. The copyright holder for this preprint (which was not certified by peer review) is the author/funder, who has granted bioRxiv a license to display the preprint in perpetuity. It is made available under aCC-BY 4.0 International license.

Cornell University Institutional Animal Care and Use Committee, protocol 2011-0099. Both rats and mice were housed in a facility with 12-h light/dark cycles and provided ad libitum access to water and chow. Temperature and humidity were checked daily to ensure that these parameters stay within appropriate ranges (20–26℃, 30–70%, respectively). *α*MHC Cre, KASH (csDN-KASH) and *Lmna*$^{N195K/N195K}$ mice have been described previously [9,19] and were back-crossed at least seven generations into a C57BL/6 line [10]. To generate *α*MHC Cre$^{+/-}$ KASH$^{+/-}$ *Lmna*$^{N195K/N195K}$ mice (*Lmna* N195K csDN-KASH), male *α*MHC Cre$^{+/-}$ *Lmna*$^{N195K/+}$ and female KASH$^{+/-}$ *Lmna*$^{N195K/+}$ mice were crossed to create experimental and control littermate mice. All mice were maintained in a uniform C57BL/6 background. *Lmna* mutant mice were provided with gel diet supplement (Nutri-Gel Diet, BioServe) to improve hydration and overall health and quality of life. To induce cardiomyocyte specific KASH-mediated LINC complex disruption, 30mg/kg tamoxifen (Cayman chemicals cat#13258) suspended in sunflower oil (Sigma cat# 88921) was injected intraperitoneally daily for five consecutive days, starting at approximately 3 weeks of age, followed by a 1 week wash out. Controls included *α*MHC Cre$^{+/-}$ KASH$^{+/-}$ *Lmna*$^{N195K/N195K}$ mice treated with vehicle and *α*MHC Cre$^{+/-}$ KASH$^{+/-}$ *Lmna*$^{+/+}$ mice littermates.

To generate *Lmna*$^{N195K/N195K}$ mice expressing cGAS-tdTomato, the previously described cGAS/MB21D1-tdTom transgenic mouse [10] was crossed into the *Lmna N195K* background to generate 3×FLAG-cGAS$^{E225A/D227A}$-tdTomato positive *Lmna*$^{N195K/N195K}$ mice within two generations. All mice were maintained in a uniform C57BL/6 background. Single cardiomyocyte data was collected at stated timepoints. Controls included *Lmna*$^{+/+}$ mice expressing the cGAS construct.

To generate the inducible, cardiac specific *Lmna* deletion mouse model (*Lmna*-cKO), αMHC-MerCreMer (The Jackson Laboratory (JAX), strain 005657) and *Lmna* floxed mice (JAX strain 026284) were crossed, as previously described [27], to obtain *α*MHC Cre$^{+/-}$ *Lmna*$^{fl/fl}$ mice. αMHC- Cre$^{+/-}$ *Lmna*$^{+/+}$ littermates served as controls (*Lmna*-cWT). All mice were maintained in a uniform C57BL/6 background. To induce lamin A/C depletion in cardiomyocytes, tamoxifen was dissolved in sunflower oil (Sigma cat# 88921) to a concentration of 30 mg/kg and injected intraperitoneally (IP) at 10 weeks of age every other day for three total injections (days 1,3 and 5). Vehicle-only injections served as additional controls. To disrupt MT in-vivo mice were also injected in between tamoxifen doses with increasing doses of colchicine (days 2,4,6,8,10 sacrifice; see concentration in Fig. 6F). PBS of equal volume was IP injected for controls. Lamin A/C depletion was validated by western blot on cardiac tissue obtained from mice at 22 days post initial tamoxifen injection, using the anti-lamin A/C (sc-376248, 1:1000) and the anti-H3 (CST #44995, 1:5000) as loading control. MT disruption in the heart was validated by western blot using anti α-tubulin (ab7291, 1:3000) and GAPDH as loading control (CST #2118S, 1:5000).

Echocardiography. Mice were anesthetized with 2% isoflurane and placed on a stereotactic heated scanning base (37°C) attached to an electrocardiographic monitor. Left ventricular structure and function were determined with a Vevo 2100 imaging system (VisualSonics) equipped with a MS550D transducer (22–55 MHz). Echocardiographic parameters were measured for at least five cardiac cycles using AM mode images. Analysis was performed using the AutoLV Analysis Software (VisualSonics) and conducted by observers blinded to the mouse genotype and/or treatment groups.

bioRxiv preprint doi: https://doi.org/10.1101/2024.02.10.579774; this version posted June 2, 2025. The copyright holder for this preprint (which was not certified by peer review) is the author/funder, who has granted bioRxiv a license to display the preprint in perpetuity. It is made available under aCC-BY 4.0 International license.

Adult rat and mouse cardiomyocyte isolation and culture. Primary adult ventricular myocytes were isolated from 8- to 12-week-old Sprague Dawley rats, or 8-9 week-old mice using Langendorff retrograde aortic perfusion with an enzymatic solution as previously described [21]. Briefly, the heart was removed from an anesthetized rodent under isoflurane and retrograde-perfused on a Langendorff apparatus with a collagenase solution. The digested heart was then minced and triturated with glass pipettes to free individual cardiomyocytes. The resulting supernatant was separated and centrifuged at 300 rpm to isolate cardiomyocytes. These cardiomyocytes were then resuspended in cardiomyocyte media (Medium 199 (Thermo Fisher) supplemented with 1x insulin-transferrinselenium-X (Gibco), 1 μg/μL primocin (InvivoGen), and 20 mM HEPES, pH = 7.4 (UPenn Cell Center)) at low density, cultured at 37 ℃ and 5% CO2 with the addition of 25 μmol/L of cytochalasin D in the media.

Active sarcomere-nuclear strain coupling. Active strain coupling was quantified by back-to-back measurement of sarcomere length contractility and nuclear deformation during electrically stimulated contractions in adult cardiomyocytes (no blebbistatin is added to allow for actin-myosin contractility). Cardiomyocytes in culture media were loaded with 4μM Hoechst and transferred to a custom-fabricated cell chamber (IonOptix) mounted on an LSM Zeiss 880 inverted confocal microscope with 63×oil 1.4 numerical aperture objective. Experiments were conducted at room temperature, and field stimulation was provided at 1 Hz with a cell stimulator (MyoPacer, IonOptix). Rod shaped cells with stable contractions were selected, and baseline sarcomere length >1.7μm, and sarcomere length strain > 10% were used as inclusion criteria. For each cell, sarcomere length contractility (SL) was first measured with a transmitted light camera (IonOptix MyoCam-S) and real time optical Fourier transform analysis (IonWizard, IonOptix). For each cell, 5 steady state and consistent SL traces were recorded in a region of interest close to the nucleus, either above or below the nucleus. The microscope was then immediately switched to fast mode Airyscan confocal and Hoechst fluorescence was imaged with 405nm laser (lowest laser power), at 11 msec/frame (91 Hz), for 5 additional steady state contractions. Initial image analysis was performed using ZEN black software for Airyscan processing, which involves signal integration from the 32 separate sub-resolution detectors in the Airyscan detector and subsequent deconvolution of this integrated signal. Stimulation times were recorded with the sarcomere contractility and nuclear imaging files for offline alignment of the traces. An average sarcomere contractility trace was generated for each cell (IonWizard, IonOptix) and exported to Matlab (Mathworks R2022b) for further alignment with nuclear deformation traces. Nuclear image analysis was performed with Arivis V4D 4.0-4.1, by a pipeline to auto-segment (Otsu) the nucleus object in each frame and export nuclear morphology parameters. Nuclear length and width traces were imported to Matlab, averaged for each cell, and aligned with the corresponding average sarcomere contractility trace (after interpolation to 91 Hz to match nuclear time trace). Sarcomere and nuclear strains were calculated by dividing the corresponding instantaneous strains by the baseline lengths prior to stimulation. For each experimental group 15-20 cardiomyocytes were recorded, from 3-4 biological replicates.

hiPSC-derived cardiomyocytes: Human induced pluripotent stem cell-derived cardiomyocytes (hiPS-CMs) were purchased from Ncardia (Nc-C-BRCM). Cells were maintained in RPMI supplemented with 10% FBS (Gibco, 16000044), 1% Penicillin-Streptomycin (Gibco, 15140122), and 2% B-27 (Gibco, 17504044). After 4 days in maintenance media, 150,000 cells were plated onto glass coverslips (for immunofluorescence)

bioRxiv preprint doi: https://doi.org/10.1101/2024.02.10.579774; this version posted June 2, 2025. The copyright holder for this preprint (which was not certified by peer review) is the author/funder, who has granted bioRxiv a license to display the preprint in perpetuity. It is made available under aCC-BY 4.0 International license.

or into 12-well culture dishes coated with 10 µg/ml fibronectin (Sigma-Aldrich, F1141) diluted in DPBS with Mg^{2+} and Ca^{2+}. Cells were then allowed to recover for 4 days in maintenance media. On day 0, cells were treated with siRNAs, against either a non-targeting control (siNT) or lamin A/C (siLMNA) at a concentration of 5nM using Lipofectamine RNAiMax transfection reagent diluted in Opti-MEM. Cells were incubated with siRNAs for 5 days total, and media was changed on days two and four. On day 4, cells were also treated with either DMSO as a control, or colchicine at a concentration of 1 µM. 24 hours after DMSO or colchicine treatment, cells were harvested for downstream analysis.

Immunofluorescence of isolated cardiomyocytes. Primary rat and mouse cardiomyocytes were fixed in 4% PFA (Electron Microscopy Sciences) for 10 min, washed three times with PBS, and permeabilized in 0.1% TritonX-100 for 10 min at room temperature. After washing twice with PBS, cells were placed in blocking buffer (1:1 Seablock (Abcam) and 0.1% TritonX-100 (Bio-Rad) in PBS for at least 1 h at room temperature, then labeled with primary antibodies (see below) for 24–72 h at 4 °C. Cells were then washed three times in PBS, then labeled with secondary antibodies in PBS at room temperature for 2–4 h. Hoechst was added for the last 10 min of secondary incubation and cells were washed twice with PBS. Stained cells were mounted on #1.5 coverslips in Prolong Diamond Antifade Mountant (Thermo Fisher) for imaging. Slides were left to cure in the dark for at least 24 h prior to imaging. hiPS-CMs coverslips were fixed in 4% PFA for 10 minutes at room temperature, then rinsed in DPBS for 5 minutes three times. Cells were permeabilized with 0.5% TritonX-100 for 10 minutes, blocked in 1% BSA in PBS-T (8mM Na_2HPO_4, 150mM NaCl, 2mM KH_2PO_4, 3mM KCl, 0.05% Tween 20, pH 7.4) and incubated with primary and secondary antibodies diluted in PBS-T + 1% BSA for 1 hour each at room temperature. Samples were counterstained with DAPI solution (Sigma, D9542) for 10 minutes at room temperature, then rinsed with PBS and stored/imaged in SlowFade Gold Antifade Mountant (Invitrogen, S36936).

Mouse tissues processing and staining. Immediately following tissue collection, hearts were washed with 1X PBS and fixed in 4% paraformaldehyde (diluted in 1X PBS) at 4°C overnight. For immunofluorescence analysis, samples were washed with PBS and flash frozen in Tissue-Tek® O.C.T. Compound (Sakura, #4583) in an ethanol bath, followed by storage at –70°C. Frozen tissue blocks were cryosectioned using an Epredia™ Microm HM525 NX Cryostat (SN: S20020277) to a thickness of 5 µm, mounted on 25 × 75 × 1.0 mm Superfrost Plus Microscope slides (Fisherbrand® #12-55015) and left to air-dry for 1 hour, followed by blocking and permeabilization with a solution of 3% BSA, 5% filtered horse serum in PBS-T (0.05% Triton-X 100 and 0.03% Tween (Sigma) in 1X PBS) for 1 hour at room temperature.

For picrosirius red staining, whole ventricles were cryosectioned at 5 µm intervals and left to air dry for at least 1 hour at room temperature and stored at -20 C. Upon processing, slides were brought to room temperature and put in Bouin's fixative (Electron Micrscopy Sciences #15990-10) set in a 56 C water bath for 15 minutes then washed 2x with milliq water. Slides were then submerged in Sirius red (Sigma #365548-5G, Direct Red 80 in 1.3% picric acid, Sigma #P6744-1GA) for 2 hours gently rocking at room temperature. Slides were washed 2x in 0.5% acetic acid then dehydrated using 95% then 100% ethanol. Finally, slides were equilibrated in xylene substitute and mounted in a xylene-based media (Leica #3801731) and imaged using ScanScope Cs2 from Aperio and analyzed using the color deconvolution macro in Aperio's analysis software package.

bioRxiv preprint doi: https://doi.org/10.1101/2024.02.10.579774; this version posted June 2, 2025. The copyright holder for this preprint (which was not certified by peer review) is the author/funder, who has granted bioRxiv a license to display the preprint in perpetuity. It is made available under aCC-BY 4.0 International license.

Immunofluorescence of cardiac tissue sections. 5-µm-thick sections were placed on glass slides and processed for immunofluorescence as described in [55] with modifications. The paraffin wax was melted and deparaffinization was achieved by 2x20 min washes in xylene (StatLab, 8400-1). Samples were rehydrated through sequential 2 min washes in decreasing concentrations of ethanol (100%, 95%, 75%, 50%) followed by ddH2O. Then, glass slides were placed in 1x reveal decloaker solution (Biocare Medical, RV1000M) and heat antigen retrieval was performed for 15 min at high pressure using a pressure cooker (Instant Pot Pro 10-in-1 pressure cooker, 8 Quart, Amazon.com). After cooling, residual reveal decloaker solution was washed off in ddH2O and a hydrophobic ring was hand drawn surrounding the sections using a Super PAP pen (Electron Microscopy Sciences, 71312). Sections were washed in 1X PBS and permeabilized with 1X PBS + 0.25% Triton X-100 for 10 min. After permeabilization, sections were thoroughly washed in 1X PBS. An additional wash in 0.1% PBST (0.1% Tween 20 in 1X PBS) preceded blocking for 1 h at RT in Blocking Solution (3% BSA (Sigma Aldrich, A7906) in 0.1% PBST).

Incubation in primary antibodies (Mouse monoclonal to α-tubulin, Sigma Aldrich T5168, clone B-5-1-2, 1:50; Goat polyclonal to desmin, R&D Systems AF3844, 1:250) was performed in Blocking Solution for 48 h, at RT, in a home-made humidified chamber. Sections were rinsed in 1X PBS, and washed 2x15 min with 0.1% PBST. Then, incubation in secondary antibodies (donkey-anti-goat IgG Alexa Fluor 647, Invitrogen A21447, 1:100; donkey-anti-mouse IgG Alexa Fluor 568, Invitrogen A10037, 1:100) was performed in 0.1% PBST for 48 h, at RT, protected from the light in the humidified chamber. Alexa Fluor 488 conjugated WGA (Invitrogen, W11261) was added during the secondary antibody incubation (1:500, final concentration 25 µg/mL). The sections were rinsed in 1X PBS and stained for 20 min with Hoechst 33342, Trihydrochloride, Trihydrate (Invitrogen, H3570, final concentration 10 µg/mL in 1X PBS). Sections were thoroughly washed in 1X PBS before mounting in Prolong Diamond Antifade Mountant (Invitrogen, P36961).

Antibodies, labels, and pharmaceuticals. Hoechst 33342 (Invitrogen, H3570); SPY650-DNA, SPY555-Tubulin, and SiR-actin (0.1µL/mL, Spirochrome); Anti-alpha Tubulin mouse monoclonal antibody, clone DM1A (1:500 abcam ab264493); Anti-KIF5B rabbit monoclonal antibody clone EPR10276(B) (1:500 abcam ab167429); Anti-Nesprin1 rabbit monoclonal antibody clone EPR14196 (1:250 abcam ab192234); Anti-Nesprin-2 (1:300, kind gift from Hodzic lab); Anti-Lamin A/C (1:1000, Santa Cruz sc376248; Anti-Lamin A/C mouse monoclonal antibody clone 4C11 (1:500, CST #4777); phospho-H2A.X (1:1000 05-636, Millipore Sigma); Anti-Desmin goat polyclonal antibody (1:250 R&D Systems AF3844), Anti-Desmin (1:1000, PA-1151113); Anti-MHY7 (1:250, DSHB BA-D5); Goat anti-rabbit IgG AF 647 (1:1000, Life Technologies, A27040); Goat anti-mouse IgG AF488 (1:1000, Life Technologies, A11001); Colchicine (1 µM in DMSO, Sigma-Aldrich); Isoproterenol (1 µM in DMSO); Blebbistatin (10 µM in DMSO, Cayman Chemical).

Image acquisition.

Live 3D imaging – resting primary cardiomyocytes: For live 3D super-resolution imaging adult rat cardiomyocytes in culture media were loaded overnight with 0.1µL/mL SPY650-DNA (Spirochrome) to label nuclei, and 10µM Blebbistatin immediately before imaging, to prevent motion artifacts. Airyscan SR (super resolution) z-stacks were acquired with Zeiss 880 Airyscan confocal microscope with 63×oil 1.4 numerical aperture objective, and 640nm laser

bioRxiv preprint doi: https://doi.org/10.1101/2024.02.10.579774; this version posted June 2, 2025. The copyright holder for this preprint (which was not certified by peer review) is the author/funder, who has granted bioRxiv a license to display the preprint in perpetuity. It is made available under aCC-BY 4.0 International license.

line in a glass bottom dish. Raw images were processed with a joint deconvolution plugin (ZEN 3.5 blue).

Live cGAS-tdTomato foci – resting primary cardiomyocytes: Live isolated cGAS-tdTomato cardiomyocytes were adhered to glass bottom dishes using MyoTak (IonOptix) and loaded with 4µM Hoechst and 10µM Blebbistatin immediately before imaging. Tile scan images were acquired at room temperature with Zeiss 980 Airyscan confocal microscope equipped with Plan-Apochromat ×20 air 0.8 numerical aperture objective. Two channels (tdTomato and Hoechst), 1 µm z-stacks tile scans were acquired, stitched and Airyscan processed using ZEN black software. Nuclei from dead or severely deformed cells, or with motion or stitching artifacts were excluded from analysis.

hiPSC-CM imaging: Z-stacks (10 µm range with voxel size of 0.035 x 0.035 x 0.5 µm) of individual nuclei were acquired on a LSM Zeiss 980 Airyscan 2 confocal microscope. A Plan-Apochromat 63×oil 1.4 numerical aperture objective was used. Tile imaging was performed under 4x zoom and 2x bidirectional averaging. The SR-4Y Multiplexing (MP) acquisition mode was used for faster parallel pixel readout. Samples were excited with the 405 nm, 488 nm and 639 nm laser lines. For nuclei selection, the Hoechst channel was used to preview images and 33.7 x 33.7 µm regions of interest (ROI) were defined to frame individual nuclei. To ensure examination of properly individualized hiPSC-derived cardiomyocytes, ROIs containing more than one nucleus were not selected for imaging. Images were Airyscan processed using the ZEN black software.

Mouse tissue sections: Imaging was performed on ≥2 myocardial areas from each of at least 3 individual sections of every animal. Areas with clear striations corresponding to longitudinally sectioned cardiomyocytes were identified through the eyepiece. Z-stacks (6x 1 µm slices, 6 µm range) were acquired on a LSM Zeiss 980 Airyscan 2 confocal microscope using a Plan-Apochromat 63xoil 1.4 numerical aperture objective. Imaging was performed under 1.7x zoom and 2x bidirectional averaging, with 0.035 x 0.035 µm pixel size. Images were acquired in Super Resolution acquisition mode at 2.0x Nyquist sampling. The 639 nm, 561 nm, 488 nm and 405 nm laser lines were used for imaging of desmin, α-tubulin, WGA and Hoechst, respectively. Images were Airyscan processed using the ZEN black software.

Image analysis. Raw images were Airyscan processed with ZEN Black software and imported to Arivis V4D 4.0-4.1 for further analysis. Dedicated V4D analysis pipeline was generated to auto segment (otsu) the nuclei from 3D z-stacks or 2D maximum intensity projection (MIP) images, and export nuclear morphology parameters, such as nuclear volume/area, length, width, and aspect ratio.

Chromatin protrusions: To determine the percentage of nuclei with chromatin protrusions for each group in primary adult mouse cardiomyocytes or mouse tissue sections, three individuals were recruited to do a blind analysis. In consistency with a recent report, nuclear were identified as bearers of chromatin protrusions when having both 1) an external outline deviating from the resting ovoid shape and 2) strong DNA condensation at the nuclear poles protruding from the expected nuclear ovoid shape, leading to nuclear deformation. In the case of the mouse tissue sections, only nuclei with a discernible outline within the z-stack were included in the analysis. A stack of 819 (in the case of primary mouse cardiomyocytes) or 441 (for mice tissue sections) maximum intensity images of Hoechst-stained nuclei from all animals was given to each individual scorer to categorize as nuclei with or without a chromatin protrusion according to

bioRxiv preprint doi: https://doi.org/10.1101/2024.02.10.579774; this version posted June 2, 2025. The copyright holder for this preprint (which was not certified by peer review) is the author/funder, who has granted bioRxiv a license to display the preprint in perpetuity. It is made available under aCC-BY 4.0 International license.

the above-mentioned criteria. The average chromatin protrusions from each animal were defined from the percentage of chromatin protrusions scored by each blinded user. The average chromatin protrusions for each group were in turn derived from the average scores of individual animals of the corresponding group.

Perinuclear enrichment in isolated CMs: Perinuclear MT or Kinesin-1 enrichment was calculated on MIP images from 3x1µm z-stacks slices (total 3µm) around the midplane of the nucleus. A perinuclear ring object was created from 0.5µm dilation of the nuclear object and subtraction of the nucleus. Nuclear pole enrichment was calculated from manually traced 2 µm wide objects for each of the long and short nuclear poles.

cGAS-tdTomato foci: cGAS foci were calculated from tile MIP images with auto segmentation (Otsu) of the nuclei from the Hoechst channel, and generation of 1 µm perimeter perinuclear ring. Fixed intensity (>600AU, or >200AU for the WT vs *Lmna* N195K cGAS-tdTomato models) and size (>0.8μm^2) thresholds for cGAS-tdTomato signal were used to segment the cGAS foci in the vicinity of the perinuclear ring. The segmented cGAS foci were used to quantify the percentage of cGAS positive myocytes. To prevent contamination by cytosolic cGAS intensity, only foci touching the Hoechst signal and originating within the 1 µm diameter perinuclear rings were included as readouts for nuclear ruptures.

γH2A.X foci: hiPSC-CMs images were analyzed with custom Arivis V4D 4.1 pipeline to segment the Hoechst channel using the Otsu method to outline the nuclei in 3D z-stacks. γH2A.X foci were segmented using fixed intensity (>2000 A.U.) and volume (0.02-1000 μm^3) thresholds. Nuclear volume and mean nuclear intensity of γH2A.X signal were calculated using the nuclear segmentation. Puncta volume, integrated volume of all intranuclear puncta, and number of puncta were calculated from the segmented γH2A.X images. The data were imported to Matlab (Mathworks R2022b) for pooling, and subsequent graphing and analysis was performed using Origin 2019 (OriginLab Corporation). The integrated volume of all γH2A.X foci within a nucleus was divided by the corresponding nuclear volume to calculate the fraction of the nuclear volume occupied by γH2A.X foci, or foci fraction of volume coverage. The integrated volume of all γH2A.X foci within a nucleus was divided by the number of foci in that nucleus to calculate the mean γH2A.X foci volume. Data points with a value greater than 3 standard deviations (SD) above the mean of the γH2A.X mean foci volume and/or of the number of γH2A.X foci were considered outliers and excluded from the analysis. These two parameters were used to identify outliers because they showed the highest variability among the measured parameters. Data were normalized to the mean of the NT DMSO group for each experimental replicate.

CM area coverage in tissue sections: Individual CMs were segmented on MIP images from 4x1µm z-stacks slices (total 4µm), using dedicated Arivis pipeline with watershed (membrane) algorithm on the WGA signal. The sum area of all segmented CMs in a single image was divided by the total image area to calculate CM area coverage.

CM nuclei and perinuclear cytoskeleton in tissue sections: Individual CM segments from CM area coverage analysis were superimposed with Hoechst channel to identify and select only CMs with fully covered nuclei and generate a mask in the Hoechst channel with only the selected CMs. The masked Hoechst channel was further background corrected and Otsu algorithm applied to automatically segment the CM nuclei. Further, PN ring segments were created from 0.5µm dilation of the nuclear segments and subtraction of the nucleus. Similarly, cytoplasmic ring segments were created from 3µm dilatation of the PN segments. Nuclear

bioRxiv preprint doi: https://doi.org/10.1101/2024.02.10.579774; this version posted June 2, 2025. The copyright holder for this preprint (which was not certified by peer review) is the author/funder, who has granted bioRxiv a license to display the preprint in perpetuity. It is made available under aCC-BY 4.0 International license.

morphology parameters were extracted from the nuclear segments, and mean fluorescence desmin and α-tubulin intensities from the PN and Cyt. segments.

Statistics. Statistical analysis was performed using Matlab, OriginPro (Version 9 and 2018), Prism (v10.1.2) and R (v.4.0.2). Statistical test and information on biological and technical replicates can be found in the figure legends. When applicable individual nuclei or cells are indicated with open circles with statistical analysis performed on the pooled data. In addition, mean values of individual animal replicates are superimposed and labeled as closed triangles, and connected by lines between the corresponding experimental conditions, for box and bar plots, the mean line is shown, with whiskers denoting standard error (SE) or the standard devition (SD) from the mean as indicated in each figure legend. Statistical tests for each comparison are denoted in the figure legends. Survival curves were generated using the Kaplan–Meier method and differences in survival tested with a Breslow test.

Computational model. The adult cardiomyocyte is rod shaped and often binucleated, with cylindrical symmetry that is largely preserved with *Lmna* mutations and LINC complex perturbations. For simplicity, we leverage this symmetry and consider an axisymmetric model covering only a quarter of the cardiomyocyte (see Fig. 7A and Table S1). Using the COMSOL multi-physics software, we simulate nuclear morphological changes due to the mutation and LINC complex disruption. Fig. 7A illustrates a typical finite element mesh used for these simulations. According to this figure, the model comprises three main parts: 1) myofibrils (cytoplasm), 2) nucleus, and 3) perinuclear MT cage, which are explained in the following sections.

Myofibrils: Adult cardiomyocytes are predominantly filled with myofibrils, encapsulated by the surrounding sarcolemma. Myofibrils are long contractile fibers that are comprised primarily of interdigitating actin and myosin filaments which are precisely held together by titin proteins [56]. Experimental observations show that during diastole, actomyosin interaction is not fully off and thus there is an active contraction in resting cardiomyocytes [34]. This contraction encounters resistance from restoring forces mediated by titin protein [34] and geometric constraints imposed by the myocardium microenvironment [33]. Our simulations (discussed later) demonstrate that this diastolic contraction laterally compresses central nuclei, causing them to elongate in the axial direction. This nuclear elongation, in turn, compresses the surrounding MT cage, generating pushing elastic forces in the perinuclear MT cage. Consequently, a complex prestress field exists in the cardiomyocyte that governs the nuclear morphology changes. The existence of this resting stress field is supported by the observation that the elongated cardiomyocyte nuclei become round upon isolation [32]. To induce this stress field in our model, we note that available experiments [57] show that myofibrils tend to assemble in the direction of maximum principal stress [37]. This implies that the myofibrillar organization during maturation is mainly controlled by stress-activated signaling pathways (like calcium pathway) [33]. Therefore, we employ our previously developed chemo-mechanical model [36] to capture this stress dependent assembly of the myofibrils. To this end, we first hypothesize an (imaginary) stress-free configuration for the cardiomyocyte, wherein the nuclei are round, and actomyosin fibers (myofibrils) are randomly distributed and not yet assembled (Fig. 7A). We further assume the following relation between the myofibrillar prestress field (σ_{ij}^{mf}), the rest

bioRxiv preprint doi: https://doi.org/10.1101/2024.02.10.579774; this version posted June 2, 2025. The copyright holder for this preprint (which was not certified by peer review) is the author/funder, who has granted bioRxiv a license to display the preprint in perpetuity. It is made available under aCC-BY 4.0 International license.

contractility (shown by tensor ρ_{ij}), and the corresponding strain field (ε_{ij}) (see [36] for more details):

$$\sigma_{ij}^{mf} = \bar{K}\varepsilon_{kk}\delta_{ij} + 2\bar{\mu}\left(\varepsilon_{ij} - \frac{1}{3}\varepsilon_{kk}\delta_{ij}\right) + \bar{\rho}_0\delta_{ij}, \tag{S1}$$

$$\rho_{ij} = \bar{\rho}_0\delta_{ij} + \bar{K}_\rho\varepsilon_{kk}\delta_{ij} + 2\bar{\mu}_\rho\left(\varepsilon_{ij} - \frac{1}{3}\varepsilon_{kk}\delta_{ij}\right), \tag{S2}$$

where, we have:

$$\bar{\rho}_0 = \frac{\beta\rho_0}{\beta - \alpha_v}, 3\bar{K} = \frac{3K\beta - 1}{\beta - \alpha_v}, 2\bar{\mu} = \frac{2\mu\beta - 1}{\beta - \alpha_v}, 3\bar{K}_\rho = \frac{3K\alpha_v - 1}{\beta - \alpha_v}, 2\bar{\mu}_\rho = \frac{2\mu\alpha_v - 1}{\beta - \alpha_v}, \tag{S3}$$

in which β is the chemical stiffness, α_v is the chemo-mechanical feedback parameter, and $K = E/(3(1-2\nu))$ and $\mu = E/(2(1+\nu))$ are the bulk and shear moduli with E and ν as the elastic modulus and Poisson's ratio, respectively. Furthermore, ρ_0 is the initial contractility which is zero in the stress-free configuration. The selected values for these model parameters are given in Tables. S2 and S3.

By increasing the initial contractility (i.e., ρ_0) (Fig. 7B), we then simulate the assembly of myofibrils, resulting in a tendency for the cardiomyocyte to shrink. However, this cell shrinkage is resisted, primarily in the longitudinal direction, by titin proteins and the geometric constraints of the cardiomyocyte microenvironment [58], as mentioned earlier. Accordingly, we restrict the longitudinal displacement of the cell at its two ends. Therefore, radial contraction occurs in the cell while its length remains fixed (see Supplementary movie S3). This constrained contraction gives rise to the generation of an anisotropic stress field inside the cytoplasm, as illustrated in Fig. 7C and Extended Data Fig. 6A. As a result of this anisotropic stress field, the contractility tensor ρ_{ij} will be no longer isotropic (see Eq. S2) since stress activated signaling pathways promote formation of myofibrils in the direction of maximum principal stress [35,37]. Extended Data Fig. 6B illustrates the obtained directions for the maximum principal stress (maximum tensile stress) field inside the cytoplasm. Notably, there is a great match between these directions and the directions of myofibrils in the physiological conditions.

Nucleus: The nucleus is primarily composed of two key mechanical components: chromatin and the nuclear envelope (NE) with its underlying lamina. The NE includes nuclear membranes, and nuclear pore complexes (NPCs), while chromatin serves as the primary building block of the nucleoplasm. Experimental observations [59] indicate that chromatin plays a crucial role in resisting small nuclear deformations, while NE and its underlying lamina predominate in scenarios involving large nuclear deformations. These experiments further suggest that the lamina network, especially lamins A and C, exhibits significant strain stiffening, whereas chromatin remains linear even at large deformations. Consequently, we model the nucleoplasm (chromatin) as a linear elastic material with a Young's modulus of 150 Pa [35] and the NE and its underlying lamina as a neo-Hookean hyperelastic layer with an elastic modulus of 15 kPa [60,61]. This hyperelastic material model captures the strain stiffening of the

bioRxiv preprint doi: https://doi.org/10.1101/2024.02.10.579774; this version posted June 2, 2025. The copyright holder for this preprint (which was not certified by peer review) is the author/funder, who has granted bioRxiv a license to display the preprint in perpetuity. It is made available under aCC-BY 4.0 International license.

NE and its underlying lamina upon large deformation. Furthermore, our in vivo observations demonstrate that relative nuclear volume changes due to lamin mutation and LINC complex disruption are less than 10 % (Fig. S6C). Based on these findings, we further consider the nucleoplasm (chromatin) to be nearly incompressible with $\nu = 0.49$, while the NE and its underlying lamina are modeled as a fully incompressible hyperelastic layer, similar to the approach in [60].

Perinuclear MT cage: In adult cardiomyocytes, microtubule organizing centers are predominantly associated with the NE [62]. MTs anchor to the NE through the LINC complex, particularly nesprin-1 proteins and kinesin motors [17], forming a dense and active cage around the nucleus. Consequently, MTs can exert active pushing forces on the nucleus through their polymerization and recruitment of the kinesin motors. This is supported by our super-resolution images (Fig. 6A), which reveal a wavy form of MTs around the nucleus, indicating that these rod-shaped fibers are under compression. To account for these active forces and for simplicity, we introduce an isotropic and homogenous compressive stress field with magnitude σ_{MT} to the perinuclear MT cage (Eq. S4). We increase this external stress from zero in the stress-free configuration to 500 Pa when inducing the prestress field under WT (physiological) conditions (Fig. 7B). Furthermore, considering the observed microtubule wavelengths, typically between 2-3 µm (Fig. 6A-B), the compressive force experienced by the MTs is estimated to be 100 pN or more [63]. This force is 10 times larger than the force of MT polymerization and 14-15 times larger than the force applied by kinesin motors [64]. Consequently, we assume that MTs are also under passive elastic compression in adult cardiomyocytes. Accordingly, we model the MT cage as an (active) linear elastic ellipsoid in the stress-free configuration. The constitutive equation for this cage can be written as:

$$\sigma_{ij}^{MT} = K_{MT}\varepsilon_{kk}\delta_{ij} + 2\mu_{MT}\left(\varepsilon_{ij} - \frac{1}{3}\varepsilon_{kk}\delta_{ij}\right) - \sigma_{MT}\delta_{ij}, \tag{S4}$$

where K_{MT} and μ_{MT} are the bulk and shear moduli of the MT cage, respectively. Fig. 7C and Extended Data Fig. 6E show the obtained stress field in the cage. According to these figures, the MT cage imposes maximum compressions on the long and short tips of the nucleus in the longitudinal (z direction) and radial (r direction) directions, respectively. This is consistent with our current observations that nuclear ruptures mostly occur at the long tips and also with our recent experimental findings [3] that MTs indent into desmin knockdown nuclei mostly around the short sides and in the radial direction.

Table S1: Geometry parameters of the model parts in the stress-free configuration.

Parameter	value
Cell radius	35 μm
Cell length	100 μm
Nuclear radius	4 μm
NE + lamina thickness	0.2 μm
MT cage long axis length	8 μm
MT cage short axis length	4.4 μm

Table S2: Material properties of the model parts.

	Young's Modulus (E)	Poisson's Ratio (ν)	Source
Cytoplasm	1.2 kPa	0.3	Typical value [19]

bioRxiv preprint doi: https://doi.org/10.1101/2024.02.10.579774; this version posted June 2, 2025. The copyright holder for this preprint (which was not certified by peer review) is the author/funder, who has granted bioRxiv a license to display the preprint in perpetuity. It is made available under aCC-BY 4.0 International license.

MT cage	1.2 kPa	0.3	Typical value
NE + lamina	15 kPa	0.5*	[13]
Chromatin (nucleoplasm)	150 Pa	0.49*	[10]

* See The main text for more details.

Table S3: Parameters for the active deformations of the cytoplasm and the MT cage.

Parameter	Value	Source
ρ_0	1.2 kPa	Estimated
α_v	2.3 kPa^{-1}	[8]
β	2.77 kPa^{-1}	[8]
MT cage active compressive stress (σ_{MT})	0.5 kPa	Estimated
