# Supplementary material for: Microtubule forces drive nuclear damage in LMNA cardiomyopathy
Source: bioRxiv. 2024 Jun 21:2024.02.10.579774. Preprint. [Version 3] doi: 10.1101/2024.02.10.579774 (PMC11212868; doi:10.1101/2024.02.10.579774)
Supplement: Supplementary file 1 [file NIHPP2024.02.10.579774v3-supplement-4.pdf]

bioRxiv preprint doi: https://doi.org/10.1101/2024.02.10.579774; this version posted June 21, 2024. The copyright holder for this preprint (which was not certified by peer review) is the author/funder, who has granted bioRxiv a license to display the preprint in perpetuity. It is made available under aCC-BY 4.0 International license.

Table S1: Geometry parameters of the model parts in the stress-free configuration.

Parameter	value
Cell radius	35 μm
Cell length	100 μm
Nuclear radius	4 μm
NE + lamina thickness	0.2 μm
MT cage long axis length	8 μm
MT cage short axis length	4.4 μm

Table S2: Material properties of the model parts.

	Young's Modulus (E)	Poisson's Ratio (ν)	Source
Cytoplasm	1.2 kPa	0.3	Typical value [19]
MT cage	1.2 kPa	0.3	Typical value
NE + lamina	15 kPa	0.5*	[13]
Chromatin (nucleoplasm)	150 Pa	0.49*	[10]

* See The main text for more details.

Table S3: Parameters for the active deformations of the cytoplasm and the MT cage.

Parameter	Value	Source
ρ_0	1.2 kPa	Estimated
α_v	2.3 kPa^{-1}	[8]
β	2.77 kPa^{-1}	[8]
MT cage active compressive stress (σ_{MT})	0.5 kPa	Estimated

bioRxiv preprint doi: https://doi.org/10.1101/2024.02.10.579774; this version posted June 21, 2024. The copyright holder for this preprint (which was not certified by peer review) is the author/funder, who has granted bioRxiv a license to display the preprint in perpetuity. It is made available under aCC-BY 4.0 International license.

Supplementary Information

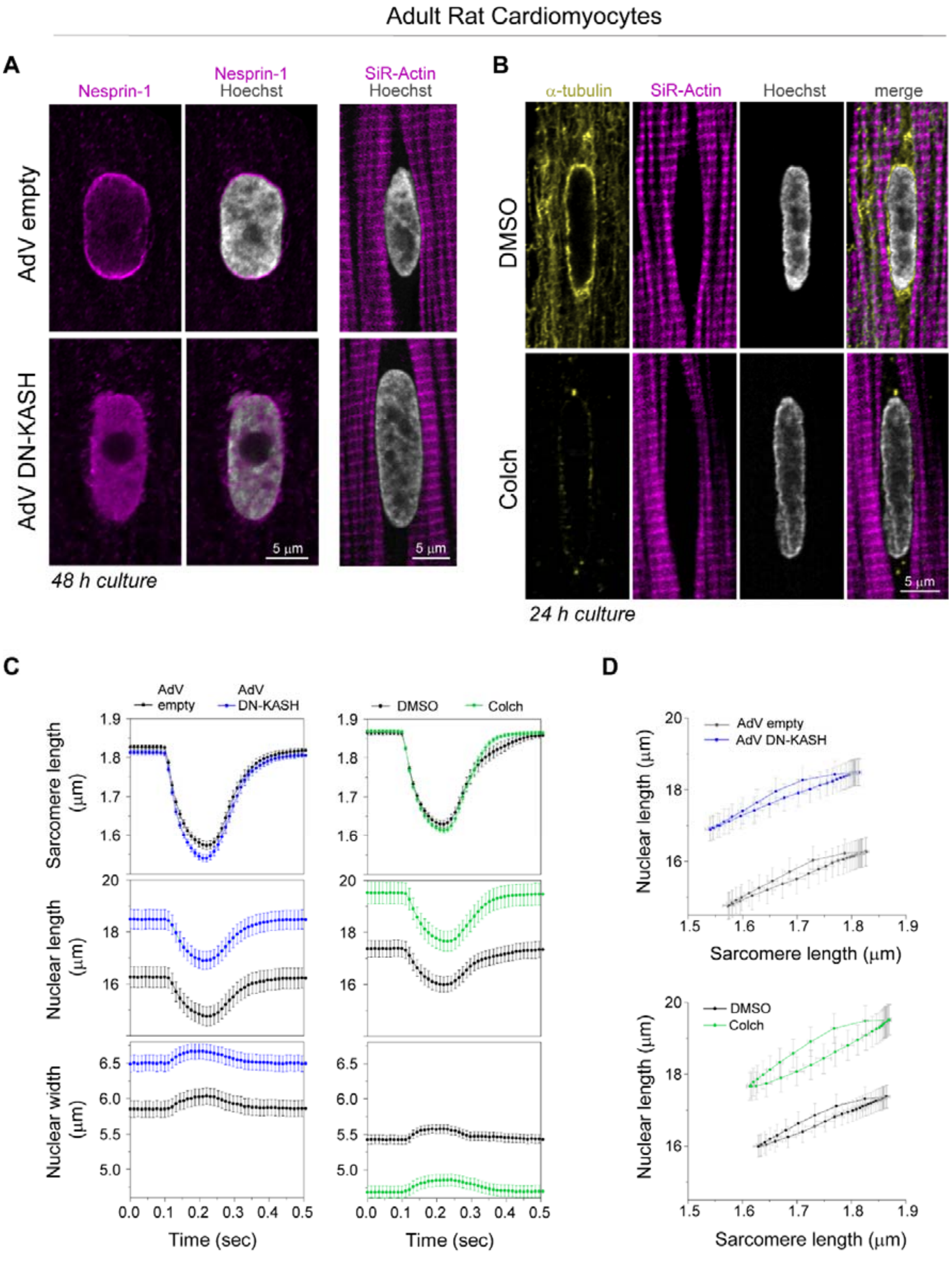

<u>Fig. S1:</u> (A) Nesprin-1 perinuclear enrichment in adult rat cardiomyocyte is lost following 48 h of adenoviral DN-KASH transduction, with no apparent changes in perinuclear sarcomeric organization (labeled with SiR-

bioRxiv preprint doi: https://doi.org/10.1101/2024.02.10.579774; this version posted June 21, 2024. The copyright holder for this preprint (which was not certified by peer review) is the author/funder, who has granted bioRxiv a license to display the preprint in perpetuity. It is made available under aCC-BY 4.0 International license.

actin). (B) MT network (yellow) is enriched at axial nuclear tips depleted of myofibrils (magenta). Colchicine treatment (1µM) for 24 h results in complete loss of the MT network (including the dense perinuclear MT cage), with no apparent change in perinuclear sarcomeric organization (labeled with SiR-actin). Representative nuclear mid-plane images are shown. (C) Sarcomere length, nuclear length, and nuclear width recordings over time during stimulated cardiomyocyte contraction. Left - cardiomyocytes treated for 48 h in culture with AdV empty (black) or AdV DN-KASH (blue). Right - cardiomyocytes treated for 24 h in culture with DMSO (black) or 1µM colchicine (green). (D) Nuclear length versus sarcomere length coupling plots for AdV DN-KASH and colchicine treatments. AdV empty and AdV DN-KASH (48 h): N=3, n=51. DMSO and colch (24h): N=3, n=51. Data presented as mean ± SE.

bioRxiv preprint doi: https://doi.org/10.1101/2024.02.10.579774; this version posted June 21, 2024. The copyright holder for this preprint (which was not certified by peer review) is the author/funder, who has granted bioRxiv a license to display the preprint in perpetuity. It is made available under aCC-BY 4.0 International license.

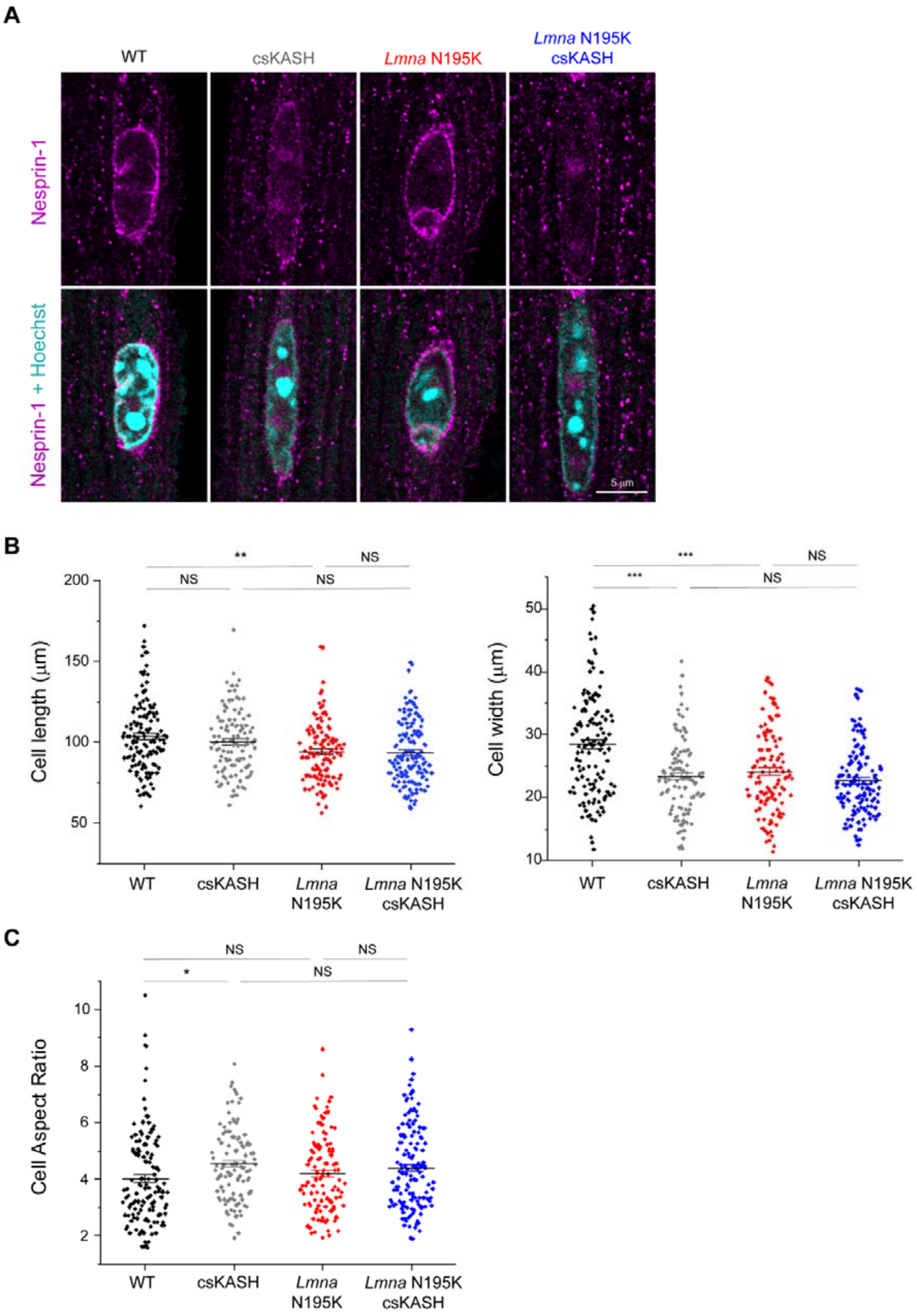

Fig. S2: (A) Nesprin-1 perinuclear enrichment in adult WT and *Lmna* N195K mouse cardiomyocytes is lost following cardiac specific in-vivo disruption of the LINC complex (csKASH). Representative nuclear mid-plane images are shown. (B) Isolated cardiomyocyte cell length, width and (C) cell aspect ratio for the indicated

bioRxiv preprint doi: https://doi.org/10.1101/2024.02.10.579774; this version posted June 21, 2024. The copyright holder for this preprint (which was not certified by peer review) is the author/funder, who has granted bioRxiv a license to display the preprint in perpetuity. It is made available under aCC-BY 4.0 International license.

mouse models. WT: N=3, n=133. csKASH: N=3, n=105. *Lmna* N195K: N=3, n=113. *Lmna* N195K csKASH: N=3, n=143. Data presented as mean ± SE. Statistical significance determined by 1-way ANOVA with Bonferroni correction (*p<0.05, **p<0.01, ***p<0.001)

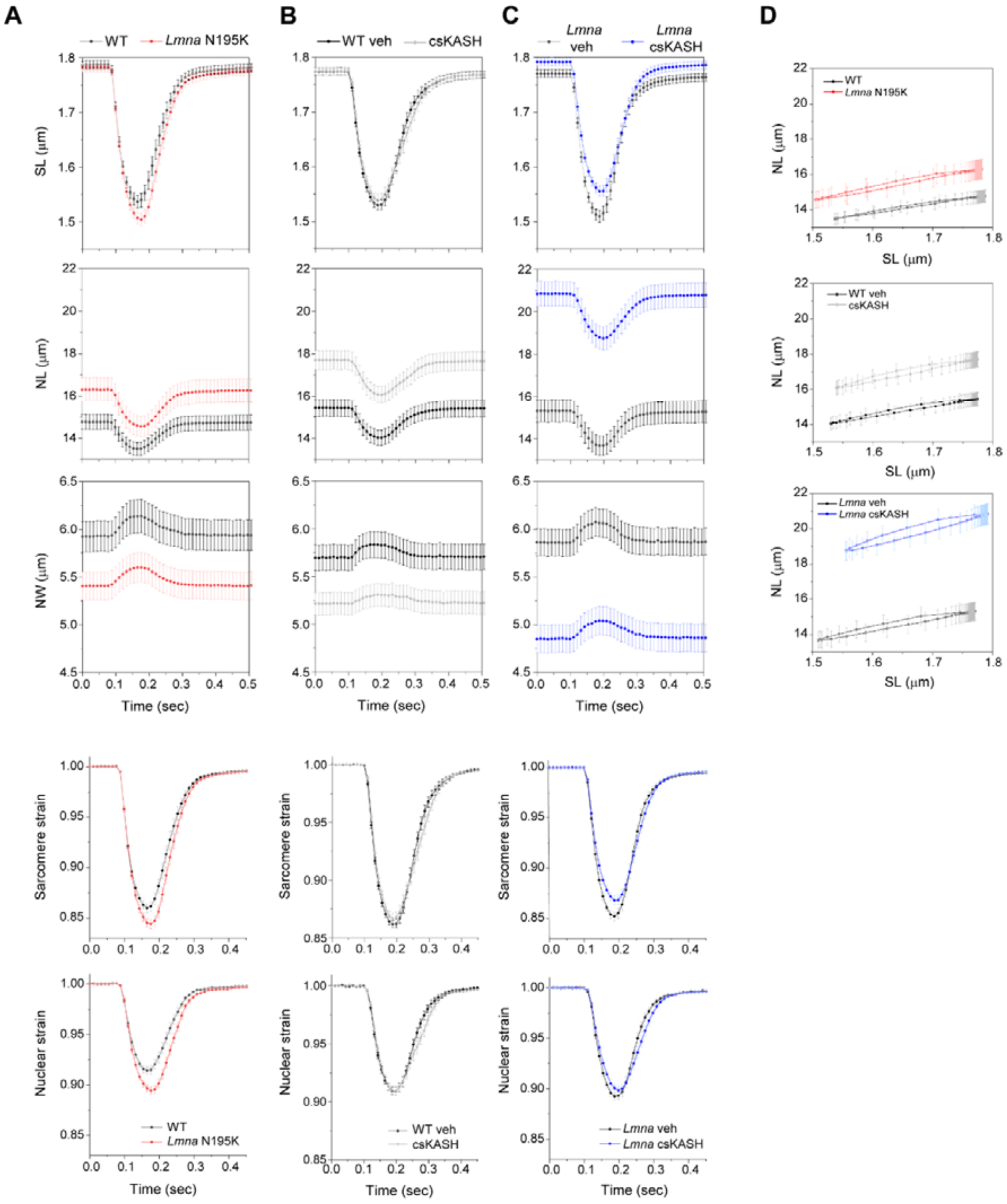

Fig. S3: Sarcomere length (SL), nuclear length (NL), and nuclear width (NW) recordings over time during stimulated contraction of freshly isolated cardiomyocytes 8-9 weeks of age. Sarcomere strain and nuclear strain over time are plotted below. (A) *Lmna* N195K (red) and WT littermate controls (black). (B) Cardiac specific LINC complex disruption (gray) and WT vehicle controls (black). (C) *Lmna* N195K with cardiac specific LINC

bioRxiv preprint doi: https://doi.org/10.1101/2024.02.10.579774; this version posted June 21, 2024. The copyright holder for this preprint (which was not certified by peer review) is the author/funder, who has granted bioRxiv a license to display the preprint in perpetuity. It is made available under aCC-BY 4.0 International license.

complex disruption (blue) and littermate *Lmna* N195K controls (black). (D) NL versus SL coupling plots for the corresponding experimental groups. WT: N=4, n=58. *Lmna* N195K: N=4, n=69. WT veh: N=4, n=81. csKASH: N=4, n=69. *Lmna* N195K veh: N=4, n=55. *Lmna* N195K csKASH: N=4, n=59. Data presented as mean ± SE.

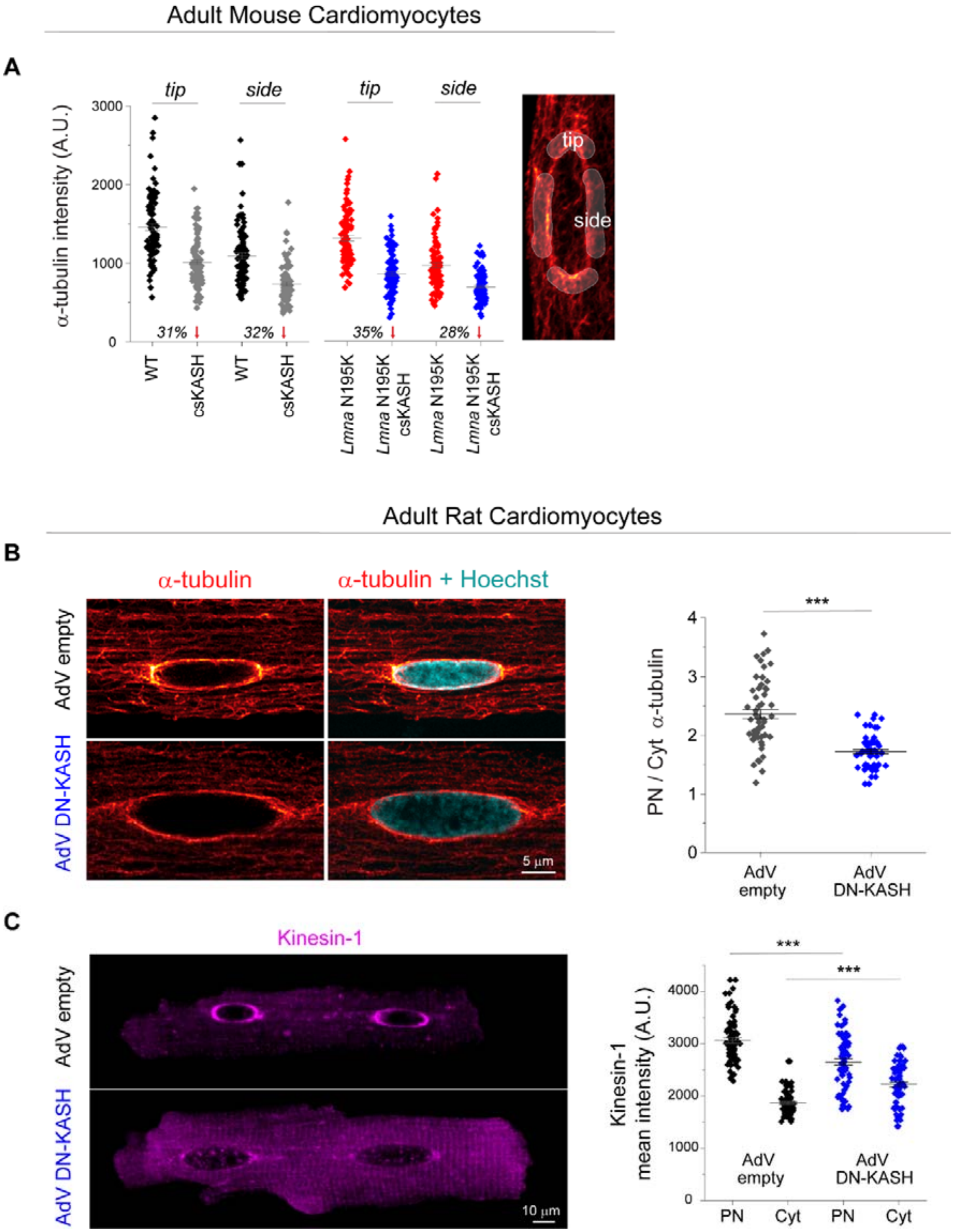

bioRxiv preprint doi: https://doi.org/10.1101/2024.02.10.579774; this version posted June 21, 2024. The copyright holder for this preprint (which was not certified by peer review) is the author/funder, who has granted bioRxiv a license to display the preprint in perpetuity. It is made available under aCC-BY 4.0 International license.

Fig. S4: (A) Quantification α-tubulin enrichment at the nuclear tips and sides in the indicated mouse model cardiomyocytes. (B) Representative nuclear mid-plane images of α-tubulin and Hoechst are shown on the left, and quantification of perinuclear α-tubulin enrichment is shown on the right. AdV empty and AdV DN-KASH: N=2, n=29. (C) Representative nuclear mid-plane images of kinesin-1 in the entire cardiomyocyte are shown on the left. Quantification of kinesin-1 intensity separately at the perinucleus and the cytoplasm is shown on the right. AdV empty: N=2, n=78, AdV DN-KASH: N=2, n=80. Data presented as mean ± SE. Statistical significance determined by two-tailed t-test (*p<0.05, **p<0.01, ***p<0.001).

bioRxiv preprint doi: https://doi.org/10.1101/2024.02.10.579774; this version posted June 21, 2024. The copyright holder for this preprint (which was not certified by peer review) is the author/funder, who has granted bioRxiv a license to display the preprint in perpetuity. It is made available under aCC-BY 4.0 International license.

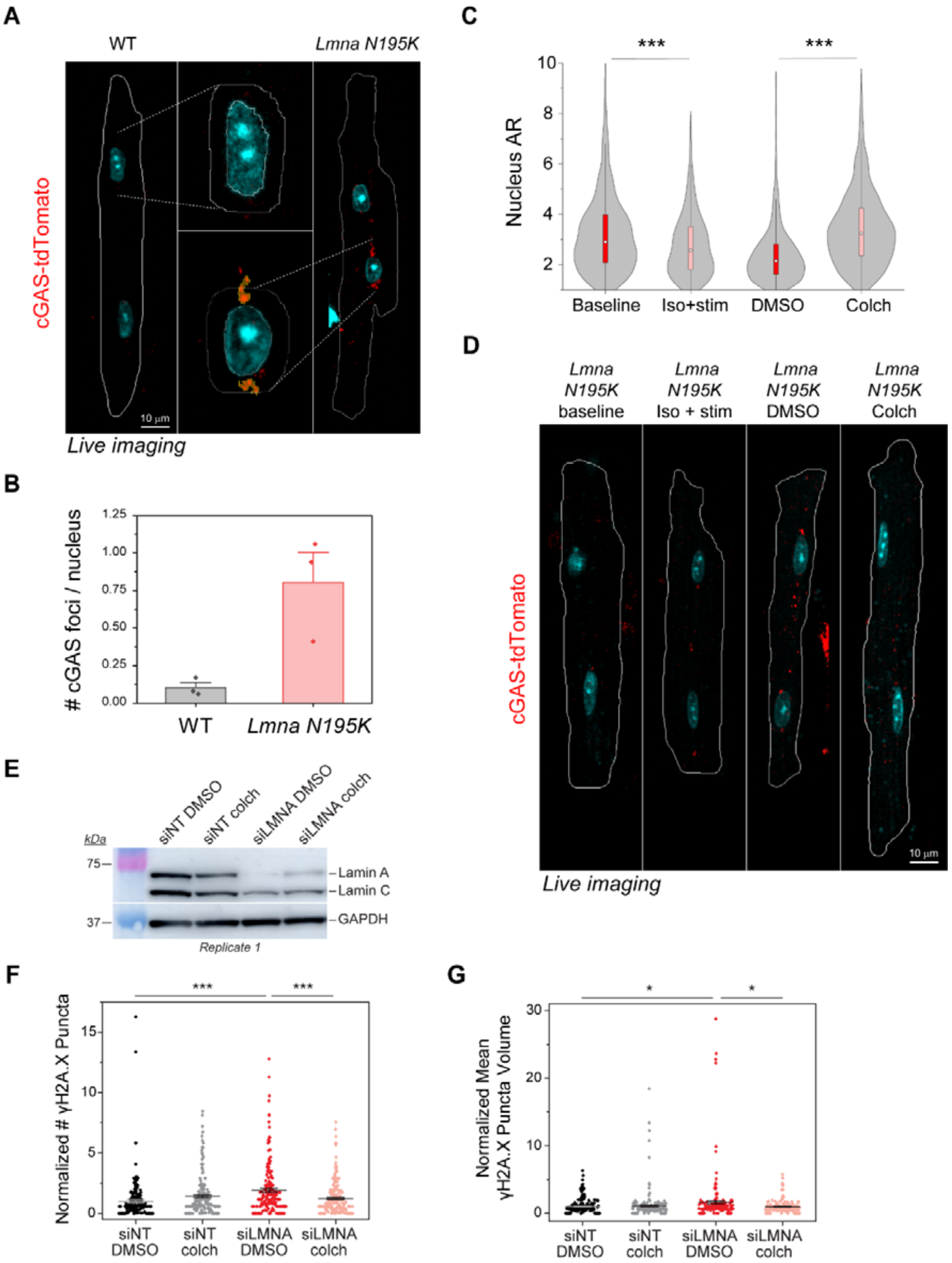

Fig. S5: (A) Full cell views of representative maximum intensity projection images labeled for nuclei (Hoechst) and cGAS-tdTomato (red), from freshly isolated cGAS-tdTomato WT, and cGAS-tdTomato *Lmna* N195K mouse models. The middle panel shows a zoom in on the nucleus and the perinuclear region used for cGAS foci quantification. (B) Quantification of the average cGAS foci number, per nucleus for WT (N=3, n=790) and *Lmna* N195K (N=3, n=381) groups. (C) Nuclear aspect ratio (AR) for the experimental groups of cGAS-tdTomato *Lmna* N195K model. N=2, n=1060 (baseline), n=780 (1 h iso + stim), n=961 (24 h DMSO), n=1141

bioRxiv preprint doi: https://doi.org/10.1101/2024.02.10.579774; this version posted June 21, 2024. The copyright holder for this preprint (which was not certified by peer review) is the author/funder, who has granted bioRxiv a license to display the preprint in perpetuity. It is made available under aCC-BY 4.0 International license.

(24 h colch). Boxplots represent the mean ± 25-75th data percentile. Statistical significance determined by two-tailed t-test. (D) Full cell views of representative maximum intensity projection images labeled for nuclei (Hoechst) and cGAS-tdTomato (red) for the indicated experimental groups. (E) Western blots from replicate 1 showing the siRNA-mediated lamin A/C knock-down in hiPSC-derived cardiomyocytes. GAPDH was used as a loading control. DNA damage induced by lamin A/C knock-down in hiPSC-derived cardiomyocytes: quantification of the number (F) and mean volume (G) of nuclear γH2A.X foci. Boxplots represent the mean ± SE. N=3, n=199 (siNT DMSO), N=3, n=197 (siNT colch), N=3, n=192 (siLMNA DMSO), N=3, n=203 (siLMNA colch). Statistical significance determined by 1-way ANOVA with Bonferroni correction (*p<0.05, **p<0.01, ***p<0.001).

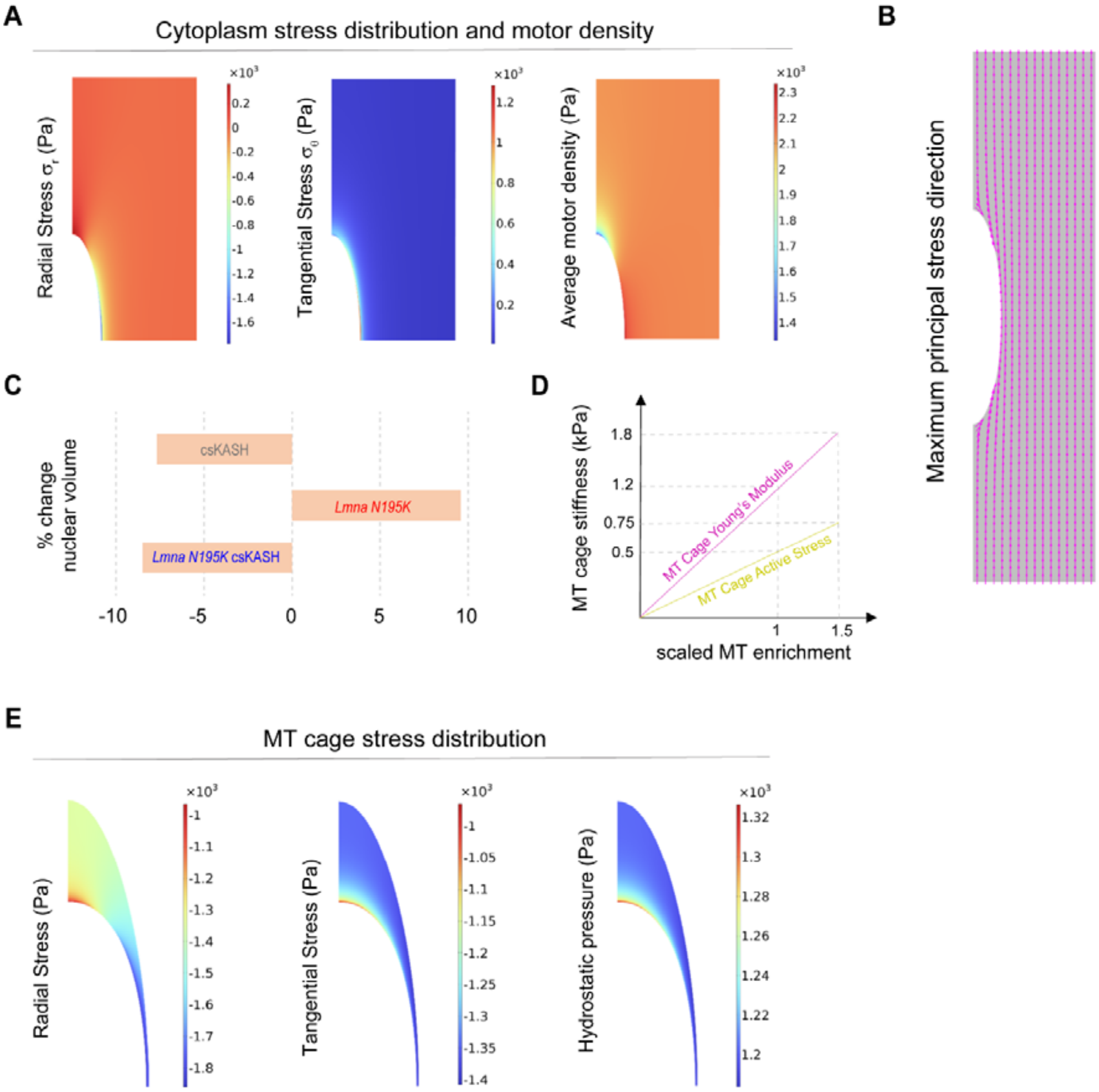

Fig. S6: (A) Simulation results depicting stress distribution and average myosin motor density in the cardiomyocyte cytoplasm under physiological conditions. Radial stress component, Tangential stress component and Average motor density defined as $(\rho_{11} + \rho_{22} + \rho_{33})/3$. (B) Model predictions for the maximum principal (maximum tensile) stress direction in the cytoplasm. (C) In vivo % volume changes relative to WT conditions.

bioRxiv preprint doi: https://doi.org/10.1101/2024.02.10.579774; this version posted June 21, 2024. The copyright holder for this preprint (which was not certified by peer review) is the author/funder, who has granted bioRxiv a license to display the preprint in perpetuity. It is made available under aCC-BY 4.0 International license.

(D) Variations of the MT cage stiffness and active stress to simulate MT enrichment around the nucleus. (E) Simulation results for stress distribution in the microtubule cage in WT conditions. Radial stress component (Pa), Tangential stress component (Pa) and Hydrostatic pressure (Pa).
